# Bio‐Inspired Soft Grippers Based on Impactive Gripping

**DOI:** 10.1002/advs.202002017

**Published:** 2021-03-02

**Authors:** Liang Zhou, Lili Ren, You Chen, Shichao Niu, Zhiwu Han, Luquan Ren

**Affiliations:** ^1^ Key Laboratory of Bionic Engineering Ministry of Education Jilin University Changchun Jilin 130022 P. R. China

**Keywords:** bio‐inspired materials, soft robotics, soft grippers, soft actuators, variable stiffness, smart materials

## Abstract

Grasping and manipulation are fundamental ways for many creatures to interact with their environments. Different morphologies and grasping methods of “grippers” are highly evolved to adapt to harsh survival conditions. For example, human hands and bird feet are composed of rigid frames and soft joints. Compared with human hands, some plants like Drosera do not have rigid frames, so they can bend at arbitrary points of the body to capture their prey. Furthermore, many muscular hydrostat animals and plant tendrils can implement more complex twisting motions in 3D space. Recently, inspired by the flexible grasping methods present in nature, increasingly more bio‐inspired soft grippers have been fabricated with compliant and soft materials. Based on this, the present review focuses on the recent research progress of bio‐inspired soft grippers based on impactive gripping. According to their types of movement and a classification model inspired by biological “grippers”, soft grippers are classified into three types, namely, non‐continuum bending‐type grippers, continuum bending‐type grippers, and continuum twisting‐type grippers. An exhaustive and updated analysis of each type of gripper is provided. Moreover, this review offers an overview of the different stiffness‐controllable strategies developed in recent years.

## Graphical abstract

According to movement ways and the classification model inspired by biological “grippers”, the soft grippers are divided into three types: non‐continuum bending‐type grippers, continuum bending‐type grippers, and continuum twisting‐type grippers. For each type of gripper, material properties, device architectures, and manipulation strategies are systematically explored and analyzed. In addition, an overview of stiffness‐controllable strategies developed in recent years is proposed.

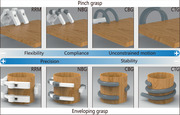

## 1 Introduction

For creatures, including both animals and plants, grasping and manipulation are essential ways to interact with their environments. Many animals and plants usually take advantage of their bodies, such as human hands, bird feet, elephant trunks, octopus tentacles, Drosera bodies, and plant tendrils, as their end effectors to achieve many interaction‐based tasks, including hunting, nest building, and feeding.^[^ ^1^ ^]^ The morphologies and functions of these end effectors have evolved in entirely different ways to adapt to environmental changes. For example, although octopus tentacles are flexible enough to implement complex twisting movements, octopuses have evolved suckers on their tentacles to ensure the stability of grasping.

Unlike the grippers of creatures, traditional robotic grippers, the earliest imitations of biological end effectors, are mainly composed of rigid frames and joints. They are widely applied in industrial robots, aeronautics, and humanoid robots, such as the famous ASIMO series. Actuators can accurately transfer power to manipulate components via traditional transmission mechanisms, such as gear and link transmission mechanisms. Traditional robotic grippers with high structural stiffness generally have the merits of a high load‐bearing capacity, fast operation speed, and precise position control. However, the high structural stiffness also limits their ability to deform elastically and adapt to target objects. More importantly, it is unsafe for humans to interact with robotic grippers with high structural stiffness; for instance, workers are not allowed to enter into the work area of robotic arms to avoid danger.^[^ ^2^ ^]^

To overcome the defects and disadvantages inherent in traditional rigid grippers, increasingly more researchers have begun to apply soft robotic technologies in the field of grippers and manipulators. These are usually called “soft grippers” or “soft manipulators,” which allow for increasing compliance and adaptability to accomplish specific tasks. The softness of robots is represented in various aspects, such as soft textures, deformable materials, elastic actuators, and soft movements, which are friendly for the manipulated object.^[^ ^3^ ^]^ Like their natural counterparts, soft grippers are compliant and flexible, and have better adaptability to various target objects. In addition, compared with traditional rigid grippers, soft grippers based on soft robotics could provide an opportunity to bridge the gap between facilities and people.^[^ ^2^ ^]^ They are characterized by improved safety, especially in scenarios that involve human interaction. For example, soft cooperative robots can be safely used in elderly and handicapped care. Additionally, soft robots for use in minimally invasive surgery inflict less damage to the human body.

Biology has long been a blueprint of inspiration for soft robotics.^[^ ^2^ ^]^ The softness and body compliance present in biology exhibit the advantage of the reducing complexity in environmental interactions.^[^ ^4^ ^]^ The material compositions, structures, and movement methods of creatures with the ability to grasp and manipulate have been studied as counterparts to soft grippers. After a long evolutionary process, the grasping methods of creatures can be roughly divided into two types, the first of which is adhesion grasping via the use of adhesives between the end effectors and target objects.^[^ ^5^ ^]^ The pangolin is a representative animal that implements this grasping method, and exploits its sticky tongue to gather white ants. The other type is impactive grasping, which is the primary grasping manner in nature. Impactive grasping includes non‐continuum bending‐type grasping represented by human hands, continuum bending‐type grasping represented by the body of the Drosera, and continuum twisting‐type grasping represented by plant tendrils. In this paper, the impactive grasping classification of creatures is equally applied to the classification of impactive soft grippers, and more details are provided in Section 3.

In addition to compliant movement methods, the softness of components is another typical feature of soft grippers. non‐continuum bending‐type grippers (NBGs) usually consist of rigid frames and soft joints, which compose a rigid‐flexible coupling system. In contrast, CBGs and CTGs consist almost completely of soft materials that play an essential role, and their material characteristics influence the manifestation of the entire system. The most frequently used compliant materials include soft silicone elastomer, polydimethylsiloxane (PDMS), and rubber, which are used as the bodies of soft grippers. The Young's modulus of these compliant materials is similar to that of the biological components of natural organisms, such as skin, muscle tissue, and cartilage, which have moduli on the order of 10^2^–10^6^ Pa.^[^ ^2^, ^6^ ^]^ This is the foremost reason why soft grippers are biologically compatible and considered to be safe for man‐machine interaction.

The addition of soft materials in the body makes soft grippers compliant and flexible. Thus, soft actuators can be integrated into the main body of grippers to allow them to implement bending or twisting motions. Different from those of traditional rigid grippers, the actuators used in soft grippers are also compliant, and can therefore deform with the grippers. In addition to the most widely used soft pneumatic actuators (SPAs) and cable‐driven actuators, the actuators of soft grippers can be made of shape memory polymers and shape memory alloys, which are temperature‐responsive. Electroactive polymers include dielectric elastomers, liquid‐crystal elastomers, and ionic polymer metal composites, which can respond to an applied electric field. Actuators can also be made of responsive hydrogels and many other new intelligent materials, which are not only compliant but also able to respond to pH, electromagnetic fields, temperature, light, and chemicals.

Soft grippers with variable stiffness have also become another research hotspot. Along with the vigorous development of soft robotics technology in recent years, many stiffness‐controllable strategies, such as the jamming effect, electrorheological fluids, magnetorheological fluids, low‐melting materials, and shape memory polymers, can be adopted in soft grippers. Soft grippers can easily switch between two states by controlling their stiffness; the low‐stiffness state has better flexibility and compliance, while the high‐stiffness state has the advantages of higher structural rigidity and a higher load capacity.

Some reviews on soft grippers have been published.^[^ ^1^, ^7^ ^]^ However, to the best of the authors’ knowledge, discussions on the development of impactive soft grippers based on a novel biologically‐inspired classification, are relatively rare. In this review, the recent developments of impactive soft grippers are discussed from a new perspective. In Section 2, inspired by creatures in nature with grasping abilities, the classification of impactive soft grippers is elaborated. In Section 3, soft grippers based on different soft actuators are reviewed. In Section 4, stiffness control strategies of impactive soft grippers are discussed. Finally, persistent challenges, future developments, and potential applications of impactive soft grippers are also proposed.

## 2 Biologically‐Inspired Manipulation Modes for Soft Grippers

**Figure 1:**
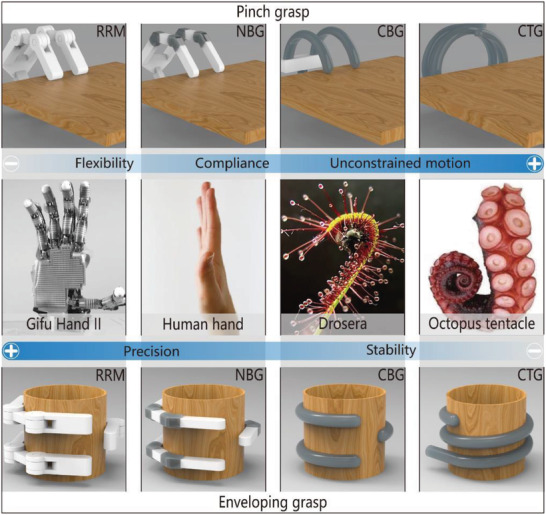
The comparison of grippers with the different driving modes, including rigid robotic manipulators (RRM), non‐continuum bending‐type gripper (NBG), continuum bending‐type gripper (CBG), and continuum twisting‐type gripper (CTG) and their typical representatives or counterparts in nature, which include the Gifu Hand II (Reproduced with permission.^[^ ^14^ ^]^ Copyright 2012, IEEE), the human hand, the Drosera, and the octopus tentacle. It should be noted that “white components” are regarded as rigid, and “gray components” are regarded as flexible.

The traditional rigid robotic manipulators (RRMs), which have rigid limbs and hinge joints, have been widely applied, especially in the fields of industrial and anthropomorphic robots. Via finite‐time control,^[^ ^8^, ^9^, ^10^ ^]^ terminal sliding mode control,^[^ ^11^, ^12^, ^13^ ^]^ and many other types of algorithms, RRMs have achieved high‐precision and high‐stability performance. As shown in **Figure** **1**, the Gifu Hand II is representative of RRMs, and is integrated with servomotors and tactile sensors that allow it to perform dexterous object manipulations.^[^ ^14^ ^]^ However, its precise position control and rigid body limit its ability to adapt to various operated objects and safely interact with humans in some scenarios, such as healthcare and cooperative human assistance.^[^ ^15^ ^]^

Compared with RRMs, the grippers of muscular hydrostat animals, plant tendrils, and boa constrictors in nature are more flexible and can adapt to contact with all kinds of shapes, surface textures, and mechanical properties. Moreover, they have an infinite number of degrees of freedom (DOFs) that allow them to bend or twist unconstrained in 3D space. Muscular hydrostat structures are found in animals, such as elephant trunks, octopus tentacles,^[^ ^16^, ^17^ ^]^ and the tongues of many animals.^[^ ^18^, ^19^ ^]^ The large and convoluted extension movements of muscular hydrostat structures, such as elongation, shortening, bending, and torsion, depend on their internal muscle fibers that are oriented in three different directions: parallel to the long axis, perpendicular to the long axis, and wrapped obliquely around the long axis.^[^ ^20^, ^21^ ^]^ Moreover, the stiffness of muscular hydrostat structures can be increased by contracting muscle or connective tissue, despite a lack of skeletal support.^[^ ^22^ ^]^ The dexterity and load‐bearing capabilities of many animals can be ascribed to their muscular hydrostat structures,^[^ ^23^ ^]^ which have also provided inspiration for the design of soft grippers. Plant tendrils are another flexible structure that can respond to variable ambient conditions and implement flexible grasping movements via bending and twisting. Their flexible physiological structure allows them to move to more profitable ecological niches with abundant sunshine and other advantages.^[^ ^24^ ^]^ Another advantage of plant tendrils is that their coiling structure can be regarded as an elastic rod, which enhances their tolerance against external impacts.^[^ ^25^ ^]^ It has been found that the internal strain mismatch within tendril tissues and the asymmetric contraction of an internal fiber ribbon with specialized cells are the leading causes of the generation of bending and twisting movements.^[^ ^26^, ^27^, ^28^ ^]^ Enlightened by the asymmetric contraction structure, many tendril‐like structures that can flexibly implement bending and twisting motions have been proposed.^[^ ^29^, ^30^ ^]^ The boa constrictor is a flexible ambush predator with an exceptional hunting pattern; it captures prey with its body, and proceeds to constrict the prey until death. Moreover, monkeys are able to keep their hands free for manipulation by using their prehensile tails to hold onto branches, and the prehensile tails of possums have the same function.^[^ ^31^ ^]^ In this paper, biological grippers with the grasping modes of muscular hydrostat animals, plant tendrils, and boa constrictors are referred to as biological continuum twisting‐type grippers (BCTGs).

The characteristics of human hands in terms of gripping are between those of traditional RRMs and tendril‐type grippers. Compared with RRMs, human hands are more flexible and compliant, whereas compared with tendril‐type grippers, they are more precise, stable, and have an improved loading capacity, which is attributed to their rigid internal structures (phalanges). As the bridge between humans and the outside world, human hands have always been an inspiration for robotic gripper design,^[^ ^32^ ^]^ which are composed of 27 bones, about 40 muscles, and more than 21 DOFs.^[^ ^33^ ^]^ They are able to perform complex and varied tasks by using an effective integrated system of mechanisms, sensors, actuators, and control functions.^[^ ^34^ ^]^ The joints of human hands have complex structures, such as ligaments and tendons, which exhibit passive compliance. The grasping modes of human hands are usually divided into two types, namely the precision grasping mode that emphasizes dexterity and sensitivity, and the power mode that emphasizes security and stability.^[^ ^35^ ^]^ Soft bionic hands driven by flexible actuators are usually designed for wrap grasping and operation in the power mode. This is because the control of the compliant structure is complicated, and the precision grasp mode is usually realized in rigid industrial grippers. In this paper, biological grippers with grasping modes similar to those of human hands are referred to as biological non‐continuum bending‐type grippers (BNBGs).

In nature, some creatures can achieve planar bending motion like human hands, whereas, in contrast to human hands, they have an infinite number of rotational DOFs in the plane. Still, they cannot twist in 3D space like muscular hydrostat animals and plant tendrils. Drosera is a typical representative of this; it is a carnivorous plant that can capture and digest insects by using sticky secretions on the surfaces of its leaves. When insects are attracted by the sweet mucilage, Drosera can rapidly bend its body to capture them.^[^ ^36^ ^]^ The bending force originates from osmotic actuation (rapid cell expansion due to an increase of internal cell pressure),^[^ ^37^ ^]^ which is capable of generating effective movements despite deficient power.^[^ ^38^ ^]^ After this, it takes hours to reset the wrapping movement.^[^ ^39^ ^]^ In this paper, biological grippers with the grasping modes like those of Drosera and other creatures that can continuously bend to achieve planar grasping motion are referred to as biological continuum bending‐type grippers (BCBGs).

In summary, impactive biological grippers can be categorized as BCTGs, BNBGs, or BCBGs. As shown in Figure 1, biological grippers include BNBGs represented by human hands, BCBGs represented by Drosera, and BCTGs represented by octopus tentacles. Their flexibility and compliance increase accordingly, while their precision and stability weaken successively.

BNBGs, which are represented by human hands, usually have rigid limbs and soft joints that provide relatively precise control and the ability to achieve stable and robust movement. In contrast, BCTGs, which are represented by octopus tentacles, have an infinite number of DOFs, which allow them to twist unconstrained in 3D space. Consequently, BCTGs exhibit the best performance in terms of flexibility and compliance. BCBGs, which are represented by Drosera, also have an infinite number of DOFs, but they often achieve only planar bending motion. Thus, the performance of BCBGs is between those of BNBGs and BCTGs. After hundreds of millions of years of evolution, these impactive grasping modes have ensured that creatures can interact with nature efficiently, and are worth investigating for application in soft robotic grippers.

In this paper, according to the proposed classification of biological grippers, impactive grippers are also divided into three categories, namely NBGs, CBGs, and CTGs (Figure 1). Moreover, the variable stiffness of soft grippers is considered.

All robotic grippers can be categorized as one of four basic types, namely impactive, ingressive, astrictive, and contigutive robotic grippers.^[^ ^40^ ^]^ Their descriptions and typical representatives are provided in **Table** **1**.^[^ ^41^ ^]^ In this paper, focus is placed on soft grippers based on the impactive gripping method. Impactive gripping usually requires the motion of solid jaws to produce the necessary grasping forces that are applied to the object from two or more directions.^[^ ^41^ ^]^ Physically grasping an object via direct impact is the most direct way to realize capture and manipulation, so impactive gripping is the most popular method used in robotic grippers.

**Table 1:** Stiffness‐controllable strategies and their features

Gripping method	Description	Typical representatives
Impactive	Physically grasp by direct impact upon the object	Jaws, clamps, and pinch mechanisms
Ingressive	Physically penetrate the surface of the object	Pins, hackles, and hook
Astrictive	Attractive forces applied to the objects surface	Vacuum suction, magnetoadhesion,and electroadhesion
Contigutive	Requiring direct contact for adhesion to take place	Chemical and thermal adhesion

The astrictive gripping method is also widely applied in soft grippers. Adhesive grippers can take the place of impactive grippers to accomplish tasks in some specific working situations, such as underwater working environments and other situations that require the grasping of objects with a smooth surface. Moreover, while the impactive gripping method can result in high surface pressures between the object and contact points, which could cause damage to the operated objects,^[^ ^41^ ^]^ the astrictive gripping method avoids this disadvantage. Many reviews on the astrictive gripping method have been conducted; for example, Jeffrey et al. and Li et al.^[^ ^42^, ^43^ ^]^ discussed dry adhesive materials and their applications, Shintake et al.^[^ ^44^ ^]^ summarized soft grippers with electro adhesion, Croll et al.^[^ ^5^ ^]^ reviewed switchable adhesives for multifunctional interfaces, and Hofman et al.^[^ ^45^ ^]^ explored underwater adhesives. The present review considers only soft grippers based on the impactive gripping method.

## 3 Soft Grippers Based on the Impactive Gripping Method

**Figure 2:**
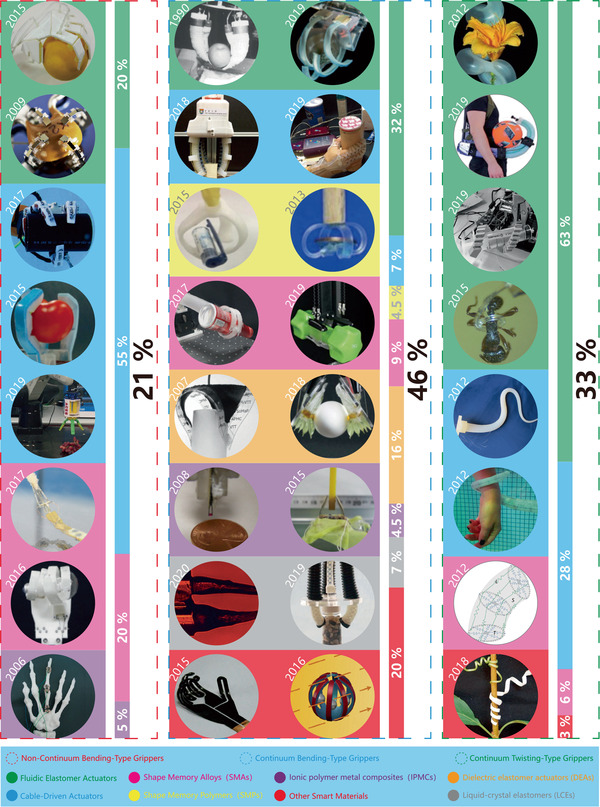
Non‐continuum bending‐type grippers (NBGs): A soft gripper with “pouch motors.” Reproduced with permission.^[^ ^46^ ^]^ Copyright 2015, Mary Ann Liebert, Inc. A soft micro‐gripper with four fingers. Reproduced with permission.^[^ ^47^ ^]^ Copyright 2009, IEEE. A cable‐driven manipulation with elastic finger joints. Reproduced with permission.^[^ ^48^ ^]^ Copyright 2017, SAGE Publications. A gripper is driven by a single cable tendon. Reproduced with permission.^[^ ^49^ ^]^ Copyright 2015, Mary Ann Liebert, Inc. An omni‐purpose soft gripper incorporated with soft fingers and a suction cup. Reproduced with permission.^[^ ^50^ ^]^ Copyright 2019, IEEE. A wireless folding gripper. Reproduced with permission.^[^ ^51^ ^]^ Copyright 2017, The American Association for the Advancement of Science. A soft hand with a good bending capacity. Reproduced with permission.^[^ ^52^ ^]^ Copyright 2016, Elsevier. A bionic finger is driven by IPMC actuators. Reproduced with permission.^[^ ^53^ ^]^ Copyright 2006, IOP Publishing. Continuum bending‐type grippers (CBGs): A flexible pressure‐driven gripper. Reproduced with permission.^[^ ^54^ ^]^ Copyright 1990, Cambridge University Press. A soft gripper is used to grasp aquatic mollusk. Reproduced with permission.^[^ ^55^ ^]^ Copyright 2019, The American Association for the Advancement of Science. A precharged pneumatic soft gripper. Reproduced with permission.^[^ ^56^ ^]^ Copyright 2018, Mary Ann Liebert, Inc. A soft wearable robot for hands. Reproduced with permission.^[^ ^57^ ^]^ Copyright 2019, Mary Ann Liebert, Inc. A gripper is driven by SMPs. Reproduced with permission.^[^ ^58^ ^]^ Copyright 2015, Springer Nature. Bidirectional SMPs. Reproduced with permission.^[^ ^59^ ^]^ Copyright 2013, John Wiley and Sons. An SMA‐based soft gripper. Reproduced with permission.^[^ ^60^ ^]^ Copyright 2017, Elsevier. A gripper with SMA springs. Reproduced with permission.^[^ ^61^ ^]^ Copyright 2019, IOP Publishing. A soft gripper based on DEME. Reproduced with permission.^[^ ^62^ ^]^ Copyright 2007, AIP Publishing. Hydraulically amplified self‐healing electrostatic gripper. Reproduced with permission.^[^ ^63^ ^]^ Copyright 2018, The American Association for the Advancement of Science. A microgripper is driven by IPMC. Reproduced with permission.^[^ ^101^ ^]^ Copyright 2008, Springer Nature. A Venus flytrap‑inspired microrobot consists of two IPMC actuators. Reproduced with permission.^[^ ^65^ ^]^ Copyright 2015, Springer Nature. A micrometer‐scale, light‐driven plier. Reproduced with permission.^[^ ^66^ ^]^ Copyright 2020, John Wiley and Sons. Electrically controlled soft gripper with three LCE tubular actuators. Reproduced with permission.^[^ ^67^ ^]^ Copyright 2019, The American Association for the Advancement of Science. A polymer electrothermal hand. Reproduced with permission.^[^ ^68^ ^]^ Copyright 2015, American Chemical Society. pH‐responsive hydrogel‐based soft micro‐robot. Reproduced with permission.^[^ ^69^ ^]^ Copyright 2016, IOP Publishing. Continuum twisting‐type grippers (CTGs): A gripper with multiple bending modes. Reproduced with permission.^[^ ^70^ ^]^ Copyright 2012, John Wiley and Sons. A wearable mobile manipulation. Reproduced with permission.^[^ ^71^ ^]^ Copyright 2019, Mary Ann Liebert, Inc. A helical soft pressure‐driven actuator. Reproduced with permission.^[^ ^72^ ^]^ Copyright 2019, Mary Ann Liebert, Inc. A soft micro‐gripper. Reproduced with permission.^[^ ^73^ ^]^ Copyright 2015, Springer Nature. Cable‐driven biomimetic gripper. Reproduced with permission.^[^ ^74^ ^]^ Copyright 2012, IOP Publishing. A soft arm inspired by the octopus. Reproduced with permission.^[^ ^75^ ^]^ Copyright 2012, Taylor & Francis. A completely soft octopus‐like robotic arm. Reproduced with permission.^[^ ^76^ ^]^ Copyright 2012, IEEE. A soft tendril‐inspired twining‐type gripper. Reproduced with permission.^[^ ^77^ ^]^ Copyright 2018, American Chemical Society.

In this section, soft grippers (referring to soft grippers based on the impactive gripping method) are discussed. **Figure** **2** presents an abridged overview of NBGs, CBGs, and CTGs, which have different features and application scenarios, just as their counterparts in nature.

CBGs account for a considerable proportion (about 46%) of soft grippers. By comparison, NBGs and CTGs respectively account for 21% and 33% of soft grippers (these statistics are based on papers referenced in this review, which were published between 1990–2020). This indicates that researchers are more inclined to use CBGs, which are characterized by simple structures, easy processing, and more convenience for the application of soft actuators.

In contrast, the manipulation modes of creatures in nature with different grasping abilities are almost all concentrated on the non‐continuum bending‐type and continuum twisting‐type modes. The continuum bending‐type grippers like those of Drosera are relatively rare in nature. This indicates that the selection mechanism of natural evolution is more inclined to grant survival to creatures with grippers whose features are more prominent. For instance, creatures with the non‐continuum bending‐type grasping mode, represented by human hands and bird feet, have the best high‐precision and high‐stability performance in nature. On the contrary, creatures with the continuum twisting‐type grasping mode, represented by plant tendrils and octopus tentacles, have the advantages of flexibility and compliance. The continuum bending‐type grasping mode is seemingly more balanced, as shown in Figure 2. Still, due to their insufficient competitiveness, creatures with this grasping mode are easily eliminated in fierce competition. However, compared with the non‐continuum bending‐type and continuum twisting‐type grasping modes, the continuum bending‐type grasping mode usually requires more simple structures and control algorithms, which is suitable for some plants like Drosera and the sporangium of the fern. All three grasping modes have different features that are worth investigating to consider their applications in soft grippers.

In nature, the BNBGs, which are represented by human hands and bird feet, usually have rigid frameworks and flexible joints covered by ligament tissue. Similar to their counterparts, the flexible joints of NBGs, which exploit the compliance of soft materials to realize bending motion similar to that of human hands, are not hinges characterized by DOFs of rotation. The compliance of joints will enhance the flexibility of the gripper and its adaptability to objects with complex geometries. Moreover, the existence of rigid frameworks allows for a stable pinch grasp. As shown in Figure 1, NBGs include another kind of grasping mode, namely enveloping grasp. The gripper can choose the proper grasping mode depending on the position, geometric shape, and size of the grasped object.^[^ ^78^ ^]^ The bending motions of soft joints are usually passive, and the joints are bent by the soft actuators, such as cable‐driven actuators. Many soft joints are flexible connections and simultaneously act as soft actuators. Compared with those of CBGs and CTGs, the stability and accuracy of the motion of NBGs are usually better due to the existence of a rigid framework, whereas their flexibility and compliance are insufficient. Moreover, due to the similarity to the structure of human hands, NBGs are an excellent choice for bionic hands for applications in the fields of anthropomorphic robots and artificial limbs.

Due to the usual movement mode of CBGs, namely continuous planar bending, which is consistent with the primary motion methods of soft actuators, almost all soft actuators can be used in CBGs. Like their counterparts, which are represented by Drosera, CBGs usually have a continuous and asymmetric structure that can be found in different types of soft actuators. This is one of the reasons why CBGs have been applied extensively in daily life. Unlike the structures of NBGs, which are integrated with rigid frameworks and compliant joints, there are typically no rigid structures in CBGs. Instead, CBGs have one or more soft and continuum fingers characterized by better flexibility and compliance. Like NBGs, most CBGs also have two kinds of grasping modes, namely pinch grasp and enveloping grasp. However, due to the absence of a rigid structure, the load‐holding capacity is relatively low compared with that of NBGs; thus CBGs are more suited to work in the enveloping grasping mode in most cases. On the other hand, the continuum structure is an obstacle to independent movement in specific parts of the finger, which is probably the greatest challenge of CBGs. Moreover, researchers have integrated some new materials and structures into CBGs to provide them with exceptional performance, such as self‐healing materials, which can provide a self‐repairing quality, an origami structure that introduces a novel driving method, and other biomimetic constructions that can significantly improve the performance of CBGs.

Twisting motion is an essential way for many animals and plants to interact with their survival circumstances. Different animals and plants wind around objects by using various patterns that allow them to reliably capture the target object.^[^ ^79^ ^]^ Twisting motions of animals and plants are common in nature, and include those by octopus tentacles, elephant trunks, the tongues of many mammals, and vines. Generally, efficient and reliable manipulation can be achieved via a twisting motion with only one gripper, which has often become the imitation object of many CTGs. Compared with NBGs and CBGs, CTGs have more advantages in terms of flexibility and compliance. They can better adapt to objects with complex geometries and achieve reliable grasping by using as few executors as possible. In fact, many CTGs only need one executor to complete a grasping job, while CBGs, as their competitors, require at least two executors to implement grasping movements. However, to achieve a twisting motion, complex structures and control algorithms are needed. The soft actuators that can only achieve bending motion may need to be redesigned for application in twisting‐type grippers. The complexity of a controllable twisting motion remains a challenge for the actuators used in CTGs. Moreover, due to their unique movement form, CTGs can only operate in the enveloping grasping mode, which could limit their applications in many use situations. For example, as shown in Figure 1, it is difficult for CTGs to grasp flat objects because they are difficult to envelop. At present, soft pneumatic actuators and cable‐driven actuators might be better choices for CTGs. Some smart materials that can generate a twisting motion via asymmetric structures have also been rapidly developed.

Generally, soft grippers need to deform or generate bending and twisting motions. Soft actuators play a crucial role in this process and are applied in different soft grippers. The applied soft grippers include soft pneumatic actuators (SPAs), cable‐driven actuators, dielectric elastomer actuators (DEAs), ionic polymer metal composites (IPMCs), shape memory alloys (SMAs), shape memory polymers (SMPs), liquid‐crystal elastomers (LCEs), and smart materials. Although actuators based on stimuli‐responsive smart materials have been developed, the traditional soft actuators like SPAs and cable‐driven actuators are still the most widely used in soft grippers. For example, cable‐driven actuators are the most widely used in NBGs. Compared with other actuators, their working pattern is more similar to that of human hands. Due to their intrinsic bending characteristics, almost all types of soft actuators could be applied in CBGs, which is why CBGs are the most popular type of soft gripper. The continuum twisting motion is more complicated than the continuum bending motion, and remains a challenge for soft actuators. More than 90% of CTGs are based on SPAs and cable‐driven actuators. While some programmable polymers and hydrogel architectures can also implement twisting motions, their movement methods are predetermined by asymmetric structures, which results in low‐flexibility control. Further, they do not provide enough gripping force to pick up heavy objects.

In this section, soft grippers based on different actuators are discussed, each of which is categorized by its manipulation mode, namely NBGs, CBGs, and CTGs. An overview of the many function parameters for soft grippers is presented in **Table** **2**. For each type of gripper, its material properties, device architectures, and manipulation strategies are systematically explored and analyzed in the subsequent subsections.

**Table 2:** The comparison of the soft actuators used in soft grippers

Category	Driving forms	Minimum response time [s]	Gripper size [cm]	Maximum load [g]	Application scenarios
SPAs	Compressed air	0.1^[^ ^80^ ^]^	0.5^[^ ^47^ ^]^–≈19.3^[^ ^46^ ^]^ ^a)^	≈131^[^ ^46^ ^]^ ^b)^	NBGs
			≈4^[^ ^81^ ^]^–20^[^ ^82^ ^]^	546^[^ ^83^ ^]^	CBGs
			0.5^[^ ^73^ ^]^–148^[^ ^84^ ^]^	89 000^[^ ^85^ ^]^	CTGs
Cable‐Driven Actuators	Pulling force	N/A	≈6^[^ ^86^ ^]^–15.8^[^ ^48^ ^]^	22 000^[^ ^87^ ^]^	NBGs
			12^[^ ^56^ ^]^–≈19.3^[^ ^57^ ^]^ ^a)^	1500^[^ ^88^ ^]^	CBGs
			25^[^ ^89^ ^]^ – 45^[^ ^90^ ^]^	65^[^ ^89^ ^]^	CTGs
SMAs	Internal energy	0.15^[^ ^91^ ^]^	≈6^[^ ^51^ ^]^–25.5^[^ ^52^ ^]^	60^[^ ^52^ ^]^	NBGs
			1^[^ ^92^ ^]^–10.078^[^ ^93^ ^]^	2000^[^ ^61^ ^]^	CBGs
			40^[^ ^94^ ^]^	478^[^ ^94^ ^]^	CTGs
SMPs	Internal energy	10^[^ ^95^ ^]^	≈0.4^[^ ^96^ ^]^–≈6^[^ ^59^ ^]^	2.5^[^ ^59^ ^]^ ^c)^	CBGs
DEAs	Electric voltage	0.096^[^ ^97^ ^]^	2^[^ ^98^ ^]^–10.5^[^ ^99^ ^]^	≈4000^[^ ^63^ ^]^	CBGs
IPMCs	Electric voltage	0.33^[^ ^65^ ^]^	6.1^[^ ^100^ ^]^–≈19.3^[^ ^53^ ^]^ ^a)^	N/A	NBGs
			0.5^[^ ^101^ ^]^–5.08^[^ ^102^ ^]^	10.3^[^ ^102^ ^]^	CBGs
LCEs	Internal energy	0.2^[^ ^103^ ^]^	0.03^[^ ^66^ ^]^ – ≈2.5^[^ ^104^ ^]^	7.4^[^ ^104^ ^]^	CBGs
			≈2^[^ ^105^ ^]^ – ≈3^[^ ^67^ ^]^	50^[^ ^67^ ^]^	CTGs

^a)^ The average length of an adult male's hand

^b)^ The average weight of a medium‐sized orange

^c)^ The weight of a penny.

### 3.1 Soft Grippers Based on SPAs

#### 3.1.1 Soft Pneumatic Actuators

In this section, soft grippers based on SPAs are reviewed. While SPAs are one of the oldest actuators, however, due to their numerous advantages, including compliance brought by the compressibility of air, safety (due to the avoidance of the use of hazardous materials, like hydraulic oil that could lead to cleanliness problems), multi‐DOF motion,^[^ ^106^ ^]^ a simple structure,^[^ ^107^ ^]^ robustness,^[^ ^108^, ^109^ ^]^ and high forces,^[^ ^110^ ^]^ they have been widely used in the field of soft grippers.^[^ ^111^, ^112^, ^113^, ^114^ ^]^ The famous McKibben artificial muscle is a representative example of the application of SPAs,^[^ ^115^, ^116^ ^]^ the performance of which is similar to that of skeletal muscle.^[^ ^117^ ^]^ Another example is a high‐force pneumatic actuator reported in 2016, whose blocked forces could reach 77.36 N at 300 kPa.^[^ ^118^ ^]^ In addition, pneumatic actuators are driven by compressed air, not high temperatures or high voltage, and are therefore safe for robotic surgery tools and other scenarios that involve human interaction.^[^ ^119^ ^]^

**Figure 3:**
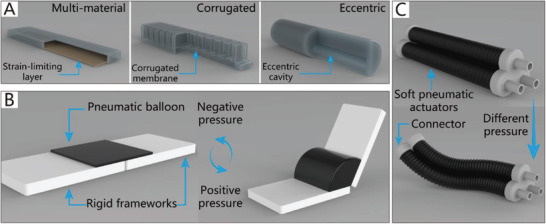
The schematic diagrams of SPAs. A) The schematic diagram of CBGs driven by SPAs. B) The schematic diagram of NBGs driven by SPAs. C) The schematic diagram of CTGs driven by SPAs.

Generally, SPAs can be roughly classified into three types based on their movement methods, the first type of SPAs includes contracting or expanding actuators (for example, Yang et al.^[^ ^120^ ^]^ reported bio‐inspired linear actuators powered by a vacuum pump; Hawkes et al.^[^ ^121^ ^]^ demonstrated an inverse pneumatic artificial muscle that attains over 300% strain and can achieve nearly linear control). The second type of SPA includes twisting actuators (for example, Kurumaya et al.^[^ ^122^ ^]^ released a modular wrist actuator for underwater manipulation; Byrne et al.^[^ ^123^ ^]^ demonstrated a twisting actuator via additive manufacturing; Yan et al.^[^ ^124^ ^]^ reported a soft torsional actuator with two spiral chambers that can generate torsional motion). The final type of SPA includes bending actuators, which are currently extensively used in the field of soft grippers. The realization of their bending motion is usually based on different types of asymmetry, including multi‐material asymmetry, corrugated membrane asymmetry, and eccentric asymmetry,^[^ ^125^ ^]^ as depicted in **Figure** **3**A. Furthermore, Figure 3B,C shows some structural asymmetry designs that could be applied in CBGs and CTGs based on SPAs.

SPAs can also be roughly categorized into three types based on their driving method, namely membrane actuators, balloon actuators, and origami actuators. Membrane actuators are systems that consist of clamped and flexible films that can expand when pressurized. For instance, Ikeuchi et al.^[^ ^126^ ^]^ developed the smallest pressure‐driven micro active catheter with a radius of 200 µm. Balloon actuators are composed of deformable chambers designed with different hollow chambers on the inside that decide the motion direction of actuators.^[^ ^127^ ^]^ One example is a gripper driven by elastomeric beams; when negative pressure is applied, a series of useful motions can be produced.^[^ ^128^ ^]^ Origami actuators are systems that consist of a flexible skin and internal origami structure, and their shape asymmetry governs the motion of the actuator. For example, an origami‐based vacuum pneumatic actuator was designed by Lee et al.^[^ ^129^ ^]^ that could produce large forces (>400 N) with a contraction ratio of >90% of the actual length.

#### 3.1.2 NBGs Based on SPAs

Typically, the pneumatic balloons of NBGs driven by SPAs simultaneously act as joints and actuators. Their working principle is illustrated in Figure 3B. The rigid frameworks are attached between pneumatic balloons as the base of the gripper like their counterpart in nature, phalanxes. The pneumatic balloons can be driven individually or collectively to meet different requirements.

**Figure 4:**
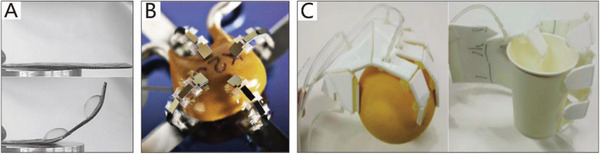
NBGs based on SPAs. A) An end‐effector driven by pneumatic balloon actuators. Reproduced with permission.^[^ ^130^ ^]^ Copyright 2001, Elsevier. B) A soft micro‐gripper with four fingers.^[^ ^47^ ^]^ Reproduced with permission. Copyright 2009, IEEE. C) A soft gripper with “pouch motors.” Reproduced with permission.^[^ ^46^ ^]^ Copyright 2015, Mary Ann Liebert, Inc.

An example is a thin and flexible end effector driven by pneumatic balloon actuators, as illustrated in **Figure** **4**B.^[^ ^130^ ^]^ The pneumatic balloon actuator is made by incorporating two layers of film that are stacked together by an adhesive to form an internal cavity. The upper film is made of silicon rubber, and the lower is made of polyimide. Consequently, when pressure is supplied, the pneumatic balloon actuator can swell toward the upper film. Similarly, the pneumatic balloon actuators simultaneously act as both actuators and joints.

Using a similar theory, Choi et al.^[^ ^47^ ^]^ proposed a soft microgripper with four fingers that consist of silicon block bases and pneumatic balloons used as joints to connect the silicon blocks. When the balloon actuator is inflated with compressed air, the attached silicon blocks will make relative out‐of‐plane motions against each other to implement a grasping motion. The microgripper can pick up various‐sized objects by changing the numbers of pneumatic balloons and silicon blocks. The microgripper has only one air inlet; thus, the four fingers can achieve the synchronization of movement. Figure 4C depicts an electric capacitor being captured by the soft microgripper at 240 kPa.

Typically, silicon rubber,^[^ ^130^ ^]^ and parylene^[^ ^47^ ^]^ are appropriate materials for pneumatic balloon actuators because their tensile strength and extensibility are sufficient to satisfy the movement demands. The manufacturing methods of balloon actuators include casting,^[^ ^131^, ^132^, ^133^ ^]^ 3D printing,^[^ ^134^, ^135^, ^136^, ^137^, ^138^ ^]^ and a large‐scale preparation method using thermoplastic film. “Pouch motors” are made first by placing two layers of films together and then hot‐pressing them using a heat‐stamping machine. With this approach, two types of drivers can be fabricated, namely the linear pouch motor (which has a maximum stroke of up to 28%) and the angular pouch motor (which has a maximum range of motion of up to 80°), which can generate expanding and contracting motions by a pneumatic control system.^[^ ^46^, ^139^ ^]^ A soft gripper based on these drivers is shown in Figure 4C.

In consideration of the compact structures of grippers, the size of the soft joints in NBGs is generally small, which has a negative impact on the power of balloon‐type actuators. For example, an extended finger based on pneumatic balloons can only produce a force of about 0.6 N on the fingertip with an air pressure of 40 kPa.^[^ ^46^ ^]^ Therefore, NBGs based on SPAs are typically used for the manipulation of small objects. Moreover, the non‐continuum structure of NBGs based on SPAs is unfavorable to the increase of the gripper length, which limits their adaptability for the manipulation of objects of different sizes. However, the non‐continuum structure introduces a competitive advantage for NBGs based on SPAs, the joints of which can be driven independently.

#### 3.1.3 CBGs Based on SPAs

Due to the security and inherent compliance of CBGs based on SPAs, they are commonly used in various industries and daily life. SPAs usually exploit differences in the extent of dilation to bend toward the given side, which can be realized by asymmetric structural designs. The common asymmetric designs and their schematic diagrams are shown in Figure 3A. The specific approaches to build CBGs based on SPAs are thoroughly discussed in this section.

**Figure 5:**
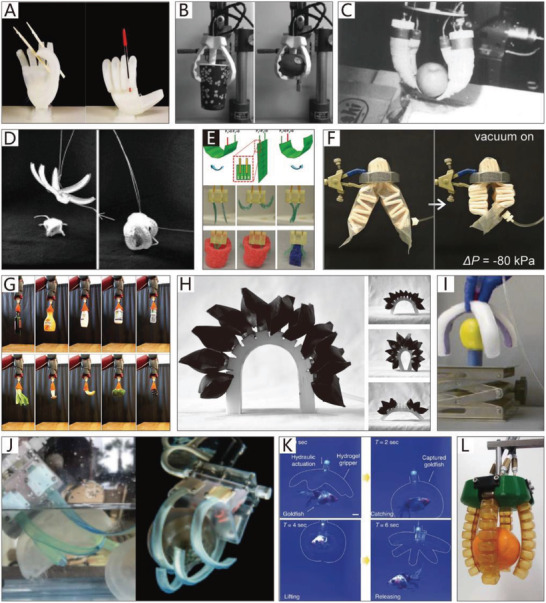
CBGs based on SPAs. A) A dexterous robotic hand driven by a PneuFlex actuator. Reproduced with permission.^[^ ^140^ ^]^ Copyright 2016, SAGE Publications. B) A bio‐inspired 3D printable soft vacuum gripper. Reproduced with permission.^[^ ^141^ ^]^ Copyright 2018, Mary Ann Liebert, Inc. C) A flexible pressure‐driven gripper. Reproduced with permission.^[^ ^54^ ^]^ Copyright 1990, Cambridge University Press. D) A starfish‐like gripper. Reproduced with permission.^[^ ^108^ ^]^ Copyright 2011, John Wiley and Sons. E) A pressure‐driven gripper with bilaterally curved fingers. Reproduced with permission.^[^ ^81^ ^]^ Copyright 2017, Mary Ann Liebert, Inc. F) A gripper with asymmetrical beam structure. Reproduced with permission.^[^ ^142^ ^]^ Copyright 2018, National Academy of Sciences – Biactive Work. G) An origami‐inspired gripper. Reproduced with permission.^[^ ^143^ ^]^ Copyright 2019, IEEE. H) A modular pressure‐driven actuator. Reproduced with permission.^[^ ^144^ ^]^ Copyright 2018, Mary Ann Liebert, Inc. I) A soft gripper fabricated by sculpting. Reproduced with permission.^[^ ^82^ ^]^ Copyright 2016, Mary Ann Liebert, Inc. J) A soft gripper used to grasp aquatic mollusk. Reproduced with permission.^[^ ^55^ ^]^ Copyright 2019, The American Association for the Advancement of Science. K) A hydraulic gripper based on transparent hydrogel actuators. Reproduced with permission.^[^ ^145^ ^]^ Copyright 2017, Springer Nature. L) A self‐healing soft pneumatic gripper. Reproduced with permission.^[^ ^146^ ^]^ Copyright 2017, The American Association for the Advancement of Science.

For instance, Deimel and Brock presented a series of dexterous robotic hands based on structural asymmetry,^[^ ^83^, ^140^ ^]^ the latest of which is driven by the PneuFlex actuator. Its bottom embodies an inelastic fabric that can prohibit the extension of rubber. Consequently, the top side is longer than the bottom side when pressurized. Thus, the actuator bends toward the bottom side. The dexterous robotic hand can be adapted to many applications, such as the manipulation of chopsticks and pens, as illustrated in **Figure** **5**A. It should be noted that, although its shape is similar to that of human hands, its working principle differs significantly.

The imitation of the structural asymmetry of the natural world has also been explored. For example, the sporangium of the fern can force its radial walls to bend via negative pressure generated in the cells.^[^ ^147^ ^]^ Using a similar driving principle, a bio‐inspired 3D printable soft vacuum actuator has been reported by Charbel et al.^[^ ^141^ ^]^ Its structural design was inspired by the sporangium of fern trees. When air is exhausted by the vacuum pump, the bio‐inspired actuator shrinks in volume and bends toward the given side. Some of the advantages of this actuator include its high actuation speed (5.54 Hz) and significant output forces (≈16 N). Based on this type of actuator, a gripper with three fingers that can pick up a cup and a kiwi fruit has been developed, as shown in Figure 5B.

The grippers described previously can only bend to one side when pressurized, and their reset motion (from the bending state to the extension state) is realized by their elasticity. Thus, the speed of the reset motion is uncontrollable. To realize controllable bilateral bending, a design has been proposed by Tedford; each motion module contains two independently‐driven chambers, and by exploiting the pressure difference between the two chambers, bilateral bending motion can be realized.^[^ ^54^ ^]^ Based on this novel design, a flexible pressure‐driven gripper that has two fingers to handle delicate fruit for export is presented in Figure 5C. Similarly, Figure 5D shows a starfish‐like gripper that has two‐layered actuators embedded in pneumatic networks of channels that can drive the actuators to bend or extend under different pressures.^[^ ^108^ ^]^ Via the use of two‐layered pouch motor actuators, Moghadam et al.^[^ ^81^ ^]^ reported a thin SPA that is hot‐pressed with four layers of thermoplastic polyurethane. As illustrated in Figure 5E(a), the first and second layers form a pouch motor, and the third and fourth layers form another pouch motor. Pressure‐driven grippers can generate movement when the two pouch motors are at different pressures. As shown in Figure 5E(b), different pick‐and‐place tasks can be achieved with bilaterally curved fingers.

Structural asymmetry in actuators can also exist in the form of an origami structure. Origami‐inspired SPAs are usually driven by a vacuum pump, which can produce large contractile strokes with relatively compact shapes.^[^ ^148^ ^]^ Moreover, the possibility of bursting and bulging, which can occur in conventional pneumatic actuators, is eliminated.^[^ ^141^ ^]^ The inner soft origami structure can improve the drive efficiency^[^ ^149^ ^]^ and provide specific bending resilience based on different folding patterns.^[^ ^150^ ^]^ Li et al.^[^ ^142^ ^]^ presented origami‐inspired artificial muscles, the performance of which is similar to or better than that of natural muscle. The peak power density of these muscles can reach 2 kW per kg when they achieve a bending motion. As shown in Figure 5F, an asymmetric beam structure was designed, which tends to the given side when the actuator is driven by a vacuum pump. Later, the authors reported another origami‐inspired vacuum‐driven gripper with a hollow hemispherical body shape.^[^ ^143^ ^]^ The gripper consists of three components, namely the origami‐based internal skeletal structure, the skin that envelopes the skeletal structure, and the connector of the gripper and manipulator. As illustrated in Figure 5G, the gripper can grasp a large variety of different‐shaped objects with sufficient gripping force.

Researchers have also been continually interested in modular pressure‐driven actuators,^[^ ^151^, ^152^ ^]^ which could overcome the existing problem of the continuum structure being unable to implement independent movement in specific parts of the actuators. One example is a modular pneumatic bending actuator fabricated by Natividad et al.,^[^ ^144^ ^]^ as shown in Figure 5H, which is composed of many removable inflatable gas cells and a flexible spine, and can act as a finger of a pressure‐driven gripper. Due to the different distributions of the pressurized flow to the modules (gas cells), the actuator can achieve different geometric variations and functions. Furthermore, the reconfigurability of the actuator allows it to rapidly be suited for different application scenarios.

From the preceding examples, it is evident that the typical working mode of CBGs based on SPAs is being driven by gas cells with structural asymmetry. The fabrication methods of gas cells are usually intricate, and include casting, 3D printing, and adhesion. To simplify the fabrication process, Argiolas et al.^[^ ^82^ ^]^ proposed a fast and simple methodology, namely the foam porous structure, to replace traditional gas cells. The foam porous structure is convenient for 3D printing and sculpting, and is therefore friendly for amateurs. Regarding the production process of foam porous structure actuators, the lost‐salt method^[^ ^153^ ^]^ is first used to fabricate foams by mixing silicone prepolymer and common salt. Then, the foams and strain‐limiting material are wrapped up together with an external seal. As shown in Figure 5I, a soft gripper fabricated by sculpting is able to grasp an apple.

The materials adopted in CBGs based on SPAs include PDMS (the elongation at break of which is 150%), Ecoflex 00–30 (the elongation at break of which is 900%),^[^ ^108^ ^]^ thermoplastic polyurethane (the elongation at break of which is 800%),^[^ ^141^ ^]^ and Ecoflex 00–10 (the elongation at break of which is 800%).^[^ ^82^ ^]^ These materials are all compliant, soft, and inherently safe, and have high elongation. They are also used in some particular fields in which the softness (elastic modulus) and self‐healing ability of materials must be considered.

The collection of fragile objects, like aquatic mollusk samples, is another application of CBGs based on SPAs.^[^ ^154^, ^155^ ^]^ Sinatra et al.^[^ ^55^ ^]^ fabricated an ultra‐gentle soft robotic gripper incorporated in a lower durometer silicone matrix (Shore 20A), which is better served to capture delicate specimens of gelatinous aquatic life. This soft grasping robot is composed of four or six slender fingers, each of which is an independent actuator that contains two layers. One of the layers is so elastic and tight that it cannot extend, while the other is flexible; as a result, the flexible layer will tend toward the tough layer under pressure. The gripper is exceptionally light (123g) and can therefore be driven using low pressure, which helps the finger to protect delicate specimens. As shown in Figure 5J, the handheld grasping device can gently grasp a moon jellyfish and a blue blubber jellyfish. To the same end, Yuk et al.^[^ ^145^ ^]^ reported a hydraulic gripper based on transparent hydrogel actuators that can be camouflaged in water and maintain their robustness and functionality over multiple cycles of actuation. The transparent hydrogel gripper can catch, lift, and release a goldfish, as shown in Figure 5K. The agile actuation and optical transparency of the gripper ensure the rate of success of capture, and due to the low stiffness of the gripper, the collected biological samples are not harmed.

Appropriate CBGs based on SPAs can protect delicate objects from damage; however, they are usually made from soft materials and are highly susceptible to damage. To overcome this limitation, Terryn et al.^[^ ^146^ ^]^ developed a self‐healing soft pneumatic gripper made of Diels–Alder polymers, as shown in Figure 5L. Via a thermo‐reversible Diels–Alder reaction,^[^ ^156^ ^]^ visible damages caused by sharp objects or the overloading of the gripper are sealed, and the initial mechanical properties are recovered. CBGs based on SPAs can also be applied in the field of soft exoskeletons. For example, Heung et al.^[^ ^157^ ^]^ developed an exoskeleton that provides convenience to the accomplishment of grasping by using a soft‐elastic composite actuator.

Furthermore, the application of SPAs in the industrial field is already quite mature, which means that they have better stability and lower fault rates. These advantages have resulted in SPA‐based CBGs being the most widely used type of soft gripper. Moreover, compared with NBGs based on SPAs, the grasping capability of CBGs based on SPAs is increased significantly, as shown in Table 2; one such gripper can lift a water bottle with a weight of 546 g.^[^ ^83^ ^]^ However, most CBGs based on SPAs cannot bend in specified locations like their counterpart in nature, Drosera. To tackle this problem, modular SPAs are a solution that could expand the application fields of CBGs based on SPAs.

#### 3.1.4 CTGs Based on SPAs

With inspiration from the typical muscular hydrostat structures of elephant trunks and octopus tentacles, many CTGs based on SPAs have been designed.^[^ ^158^, ^159^, ^160^, ^161^, ^162^, ^163^, ^164^ ^]^ They are usually driven by actuators of pressure‐driven antagonistic actuation, which consist of two or more antagonistic units; the schematic diagram is shown in Figure 3C. The difference in the gas pressure in antagonistic units forces the actuators to implement specific movements, such as bending movements^[^ ^165^, ^166^ ^]^ and rotational movements.^[^ ^167^ ^]^

**Figure 6:**
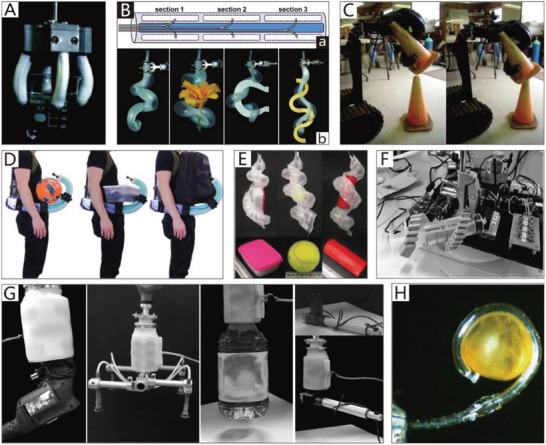
CTGs based on SPAs. A) A 4‐finger gripper. Reproduced with permission.^[^ ^169^ ^]^ Copyright 1992, IEEE. B) A gripper with multiple bending modes. Reproduced with permission.^[^ ^70^ ^]^ Copyright 2012, John Wiley and Sons. C) OctArm. Reproduced with permission.^[^ ^85^ ^]^ Copyright 2006, SPIE. D) A wearable mobile manipulation. Reproduced with permission.^[^ ^71^ ^]^ Copyright 2019, Mary Ann Liebert, Inc. E) A helical inflatable gripper. Reproduced with permission.^[^ ^172^ ^]^ Copyright 2015, IEEE. F) A helical soft pressure‐driven actuator. Reproduced with permission.^[^ ^72^ ^]^ Copyright 2019, Mary Ann Liebert, Inc. G) A high‐load soft gripper. Reproduced with permission.^[^ ^79^ ^]^ Copyright 2019, Mary Ann Liebert, Inc. H) A soft micro‐gripper. Reproduced with permission.^[^ ^73^ ^]^ Copyright 2015, Springer Nature.

As early as 1991, a flexible micro‐actuator was designed by Suzumori et al.,^[^ ^168^, ^169^ ^]^ which has three internal chambers. The micro‐actuator is reinforced with inextensible fiber in the circular direction, and it therefore easily deforms in the axial direction. By controlling the internal pressure of the three chambers, the actuator can implement pitching, yawing, and stretching movements. As presented in **Figure** 6A, a flexible soft gripper has been designed by using three such micro actuators, which can flexibly manipulate a beaker filled with liquid. Using similar actuators, Bartow et al.^[^ ^84^ ^]^ presented a continuum manipulator driven by artificial muscles connected by zip ties. By controlling the internal pressure of the artificial muscles, the manipulator can conduct different active motions. Besides, Elsayed et al.^[^ ^170^ ^]^ determined the optimal design of a pneumatically actuating silicone module via finite element analysis. Various pneumatic chamber designs for the pneumatic actuator were investigated, including chambers with a circular cross‐section, semicircular cross‐section, circular sector cross‐section, and ring sector cross‐section. Besides, the interior material of the chambers can also be optimally designed, just like a vacuum‐powered SPA, which is based on three antagonistic chambers consisting of an off‐the‐shelf foam core and brushed‐on layers of silicone rubber. The foam core chambers are more robust to external disturbance than traditional air chambers, and they can be easily fabricated without a mold or 3D printer.^[^ ^171^ ^]^

Based on the antagonistic actuation principle, the motion chambers of a typical biomimetic muscle hydrostat gripper usually influence each other, which is not conducive to the operability of soft grippers. More complex motion can be achieved by dividing the entire gripper into several parts to operate them separately. One representative example is a soft manipulator for minimally invasive surgery. Like octopus tentacles, the soft manipulator is composed of soft materials and driven by two identical modules with multi‐directional bending and stiffening capabilities. A single module can conduct active motion while the other module stiffens. The independent motion of the modules can enhance the manipulability of the soft manipulator during surgery.^[^ ^173^ ^]^ Also, based on the modular design idea, Martinez et al.^[^ ^70^ ^]^ developed a biomimetic gripper with 3D mobility that has three different pressure‐driven sections, each of which has three independent microchannels for antagonistic actuation, as shown in Figure 6B(a). The range of motion and the capability for complex manipulation increase with the number of pressure modules. As shown in Figure 6B(b), a soft gripper with multiple bending modes can adopt complex shapes and manipulate delicate objects. Another example of modular manipulators is a soft robot called OctArm that consists of three sections. Each section has individual air muscle extensors with control channels that provide two‐axis bending and extension. The soft robot manipulator can provide 890 and 250 N of vertical and transverse load, respectively, using 8.27 bar of air pressure. As shown in Figure 6C, the modular design allows the manipulator to easily meet various complex requirements.^[^ ^85^, ^174^ ^]^ Similarly, Nguyen et al.^[^ ^71^ ^]^ developed a fluid‐driven, wearable mobile manipulator for daily living tasks. The wearable manipulator equips the user with an additional limb that is safe and compliant, enabling the user to achieve complex 3D motion in space. It consists of three modular tapered segments, each of which is made of three chambers. By controlling the internal pressure of the chambers, the manipulation equipment can implement specific movements. The end‐effector functional module is also reconfigurable according to different grasping tasks, as presented in Figure 6D.

Even without a muscular hydrostat, some creatures (like boa constrictors and vines) can achieve grasping movements similar to those of octopus tentacles and elephant trunks. Many pressure‐driven manipulators inspired by the winding behaviors of these creatures have been designed.^[^ ^175^, ^176^, ^177^ ^]^ One example is a tendril‐like gripper based on an inflatable origami‐based actuator, which is constructed by connecting a soft chamber to an origami rotational joint. The advantages of the origami structure are that the soft chamber is protected from punctures, and stability is provided for the whole gripper.^[^ ^178^ ^]^ Figure 6E depicts an extremely lightweight helical inflatable gripper that consists of an inflatable soft actuator with pleated structures. While this end effector weighs only about 40 g, it has a gripping force of up to 15 N. The bag structure of the gripper consists of two upper pleated plastic‐films and one lower plastic film. Due to its asymmetry, when the bag structure is pressurized, the gripper can generate a helical motion.^[^ ^172^ ^]^ Based on the asymmetry of the structure, Hu and Alici developed a bio‐inspired helical soft pressure‐driven actuator that can simultaneously generate bending and twisting motions (Figure 6F). The pressure‐driven actuator is composed of a passive lower layer and an upper active layer with the same helix angle arranged in a row. When the actuator is under pressure, the expansion of the upper active layer is greater than that of the lower layer. The asymmetry of the upper active layer and the lower passive layer forces the gripper to move vertically and horizontally.^[^ ^72^ ^]^ Inspired by the winding behaviors of animals and plants, Li et al.^[^ ^79^ ^]^ reported four types of high‐load soft grippers, namely the spiral interleaving bionic gripper, parallel interleaving bionic gripper, spiral winding bionic gripper, and parallel winding bionic gripper, all of which are driven by pneumatic artificial muscles. A simulation and experimental analysis revealed that the parallel winding bionic gripper could achieve the highest gripping force under the same air pressure. The high‐load soft gripper can lift heavy objects that weigh up to 20 kg. As shown in Figure 6G, in addition to their higher load capacity, they can grip multiple kinds of objects with different shapes and stiffnesses. In addition, Paek et al.^[^ ^73^ ^]^ proposed a soft microgripper with spiral bending capability, which is composed of a PDMS microtube (with an inner diameter of 100–125 µm) as the platform structure and a hump as an additional structure to help the microgripper produce a tentacle‐like spiraling motion. The grasping force of the microgripper is about 0.78 mN, and it can wind around and hold fragile micro‐objects with a final spiral radius of ≈200 µm. A Mallotus villosus egg can be comfortably held by the microgripper, as shown in Figure 6H.

Based on the preceding analysis, the SPAs applied in CTGs can be divided into two categories. The first includes antagonistic actuators that consist of two or more (usually three) antagonistic units. After years of continuous improvement, modular design has been introduced to antagonistic actuators; the entire gripper is divided into several parts and meets the demand of individual operation. The manipulability and flexibility of these actuators have been improved at the expense of the complexity of control. The second type of SPA can generate twisting motions by using its structural asymmetry. While these actuators usually have compact and simple structures, their movement forms are relatively singular, which restricts both their controllability and functionality. Future studies on the application of SPAs in CTGs could focus on ensuring the flexibility and manipulability of the actuators without sacrificing their compact and simple structure.

The materials adopted in CTGs based on SPAs include Ecoflex 00–30 (the elongation at break of which is 900%),^[^ ^70^ ^]^ Dragon Skin 30 (the elongation at break of which is 364 %),^[^ ^71^ ^]^ FilaFlex (the elongation rate of which reaches 400%),^[^ ^72^ ^]^ and PDMS (the elongation at break of which is 150%).^[^ ^73^ ^]^ Materials with high elongation are a necessary qualification for CTGs based on SPAs, which must implement grasping tasks via the large deformation of soft actuators. Moreover, some measures have been taken to sufficiently enhance deformation (which usually refers to axial deformation). For example, plastic rings are inserted along the length of the actuator to restrict radial expansion.^[^ ^71^ ^]^

The continuity of CTGs is beneficial for the arrangement of powerful SPAs, which can increase the load capacity of grippers. As shown in Table 2, their length is increased, and one SPA is as long as 148 cm.^[^ ^84^ ^]^ While the increase of the gripper length makes the workspace larger, it also enhances the adaptability of the CTG to objects with different scales. However, due to the continuity of the actuators, the motion of any part of a CTG based on SPAs is dependent; thus, the separate control of a portion of the gripper is difficult.

### 3.2 Soft Grippers Based on Cable‐Driven Actuators

#### 3.2.1 Cable‐Driven Actuators

Due to the compressibility of gas, the pneumatic system is considered to be nonlinear.^[^ ^179^ ^]^ Therefore, it is challenging for pneumatic actuators to achieve accurate control.^[^ ^180^, ^181^ ^]^ In contrast, it is much easier for inextensible cables to achieve the precise control of position and force, which allows them to meet high requirements, such as those of surgical robots.^[^ ^182^ ^]^ Moreover, end effectors, such as cable‐driven hands, can be installed far from the power source by cable transmissions,^[^ ^183^ ^]^ which lowers the moment of inertia of the end effector.^[^ ^184^ ^]^ Additionally, flexible and compliant cables have a very high tensile strength along their longitudinal axis; thus, they can easily fit into the manipulators.^[^ ^1^ ^]^ Based on these advantages, many traditional cable‐driven hands consisting of rigid links and joints have been developed.^[^ ^185^, ^186^, ^187^, ^188^, ^189^, ^190^, ^191^ ^]^ Recently, cable actuators based on soft materials have been widely developed in the field of light weight soft robotic hands.^[^ ^7^, ^192^ ^]^ Due to the similarity between cable actuators and human hands regarding their driving form, cable actuators are suitable for use in the field of bionic soft hands.

However, one of the limitations of cable‐driven actuators is their elasticity, which usually adversely influences their static and dynamic performance. Additionally, routing systems that guide the cables from the power source to the end effector will add additional friction to the system.^[^ ^183^ ^]^

#### 3.2.2 NBGs Based on Cable‐Driven Actuators

**Figure 7:**
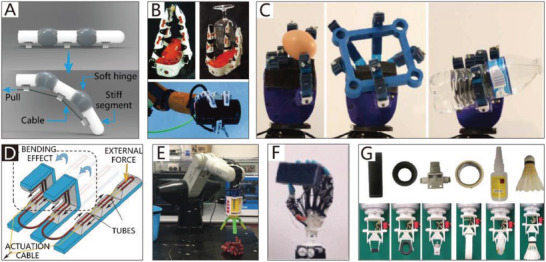
NBGs based on cable‐driven actuators. A) The driving type of the NBGs based on cable‐driven actuators. B) A cable‐driven manipulation with elastic finger joints. Reproduced with permission.^[^ ^48^ ^]^ Copyright 2017, SAGE Publications. C) A dexterous tendon‐driven hand. Reproduced with permission.^[^ ^195^ ^]^ Copyright 2019. D) A gripper is driven by a single cable tendon. Reproduced with permission.^[^ ^49^ ^]^ Copyright 2015, Mary Ann Liebert, Inc. E) An Omni‐purpose soft gripper incorporated with soft fingers and a suction cup. Reproduced with permission.^[^ ^50^ ^]^ Copyright 2019, IEEE. F) A biomimetic cable‐driven hand. Reproduced with permission.^[^ ^197^ ^]^ Copyright 2016, IEEE. G) An origami‐inspired soft gripper. Reproduced with permission.^[^ ^86^ ^]^ Copyright 2019, IEEE.

Unlike traditional rigid cable‐driven grippers, which include fingers consisting of many rigid joints between stiff segments, NBGs based on cable‐driven actuators have no rigid joints, which are instead replaced by hinges made of elastic materials. The elastic hinges introduce compliance to the system, and are also used as actuators to return the actuated fingers previously driven by cables.^[^ ^193^, ^194^, ^195^ ^]^ The detailed schematic diagram is shown in **Figure** **7**A. Based on this method, Stuart et al.^[^ ^48^ ^]^ reported an adaptive cable‐driven manipulation design that utilizes elastic finger joints and a spring transmission to implement multiple types of motions, including various pinches and grasps, like human hands. The gripper is actuated by cables, which pass over joints molded by urethane flexures and return the actuated fingers by preloaded stainless‐steel springs. The grasping ability of the gripper, which can grasp various irregularly‐shaped objects, is presented in Figure 7B. Furthermore, a dexterous tendon‐driven hand that possesses five anthropomorphic, adaptive fingers has been reported. The fingers can smoothly accomplish adduction/abduction and flexion/extension.^[^ ^195^ ^]^ As shown in Figure 7C, the bionic hand can perform various grasping tasks in daily life. Similarly, Tavakoli et al.^[^ ^196^ ^]^ reported a low‐cost, adaptive, and low‐weight (only 280 g) anthropomorphic hand that is integrated with a twisted string actuation system, which includes motors and twisted string that can convert the rotational motion of actuators into linear motion. Furthermore, the natural compliance of the system ensures that the hand can operate safely around humans.

Each finger of a soft gripper driven by cable‐driven actuators usually has an independent actuator to implement coordinated manipulation. However, the control system will become complicated when each finger is separately driven. Thus, Manti et al.^[^ ^49^ ^]^ put forward a more straightforward control method in which only a single cable tendon is used to drive the whole gripper, which has the advantages of simple control and adaptive grasping. The gripper has three fingers driven by a single cable inside the tubes. As shown in Figure 7D, when external resistance is applied to one finger, the other two fingers can still achieve the complete closure of the object to realize a full grasp. Hence, the control scheme guarantees the ability to grasp objects with different shapes. When the gripper is ready for the next grasping task, the cable will release, and the fingers will spring back.

A combination of tendon‐driven actuators and other auxiliary structures could achieve better‐grasping performance. One representative example (Figure 7E) is an omni‐purpose soft gripper that incorporates soft fingers and a suction cup, which helps to grasp a wide variety of objects with smooth surfaces. The three tendon‐driven soft fingers of the gripper are driven by linear soft vacuum actuators manufactured using low‐cost 3D printing. The soft gripper can be mounted on a 6‐DOF robotic manipulator, which is responsible for controlling the position of the soft gripper in space.^[^ ^50^ ^]^

The working pattern of NBGs based on cable‐driven actuators is quite similar to the working pattern of human hands, which have a driving system consisting of skeletal muscles, tendons, and fingers. The fingers can achieve flexing and extension, as well as abduct and adduct motions under the drive of the skeletal muscles of the palm and forearm via the delivery of tendons connected with the phalanxes. Therefore, researchers have investigated human hands with the intention of replicating their functions by robots performing tasks. Xu and Todorov developed a biomimetic cable‐driven hand with a highly biomimetic design, including the morphology of bones, tendons, and joints as the connection between two adjacent bones. As shown in Figure 7F, this hand can perform the in‐hand manipulation of a whiteboard eraser. With the aim of preserving the structural features of bones to the greatest extent, the manufacturing method of 3D printing is adopted. The adjacent 3D‐printed bones are connected by crocheted ligaments and laser‐cut joint soft tissues, which are used to mimic human soft tissues to provide similar functions as the finger joint. Ten dynamic servos are also used to imitate important large skeletal muscles, and high‐strength strings are used to imitate tendons.^[^ ^197^ ^]^

In addition to the grasping task, bionic hands can be used for the biomechanical study of human hands. To better understand human hands, another highly biomimetic tendon‐driven hand was designed by Çulha and Iida. The passive compliance of the cable‐driven hand is achieved by covering two types of elastic ligaments, including capsule ligaments, which are a layer to keep the joints in place and that slide over each other, and collateral ligaments, which surround the sides of the capsule ligaments to guide the motion of the joints. Moreover, the structural parameters of the right‐hand bones provided by 3B Scientific GmbH are adopted for anatomic accuracy. Finally, the cables are used to drive the bones of the fingers to surround the bone tips.^[^ ^198^ ^]^

In addition to the human hand‐type frame, the origami structure is another good option for the frame of cable‐driven grippers. These structures have rigid frames and relatively soft joints (the creases used for folding in the origami structure). Kan et al.^[^ ^86^ ^]^ designed an origami‐inspired soft gripper that can generate grasping movements depending on the geometric constraints of the origami spring structures. The soft gripper consists of two origami fingers and a tendon‐based actuator. The gripper is powered by the tendon‐based actuator and can be flexibly switched between a flat‐sheet mode and a gripping mode. As illustrated in Figure 7G, a prototype of a soft gripper based on the origami pattern was fabricated using elastic material via 3D printing technology, and can hold various common objects.

Different from NBGs based on SPAs, the joints of which also are actuators, NBGs based on cable‐driven actuators have passive‐compliance joints, and their elastic constraints are provided by bionic ligaments. In addition, power is provided independently by cable‐driven actuators, which can be installed in a location other than in the gripper. Thus, the increased volume and weight introduced by the high‐power actuators will not affect the operating efficiency of the manipulators. Moreover, due to the existence of rigid structures, NBGs based on cable‐driven actuators can achieve high‐stability performance; as shown in Table 2, they have the advantages of a high load capacity, high stability, and reliability.

NBGs based on cable‐driven actuators have been gradually matured in recent years. They are made with elastic materials (such as urethane,^[^ ^48^ ^]^ silicone,^[^ ^49^ ^]^ and black nitrile rubber^[^ ^198^ ^]^) and have hinges and cable tendons (made of materials such as nylon cable ^[^ ^49^ ^]^ and fishing line^[^ ^50^ ^]^) like human hands, and can smoothly accomplish various pinching and grasping tasks. Due to the material attributes of cable tendons, the deformation of which is extremely low, the response time of these grippers can often be ignored. However, a potential limitation of NBGs based on cable‐driven actuators is their load capacity. Compared with human hands, almost all NBGs can only be used under low‐load conditions, even though their driving mode and structural features are similar to those of human hands. Besides, the use of bionic NBGs based on cable‐driven actuators to study the biomechanical properties of human hands will be a potential research issue in the future.

#### 3.2.3 CBGs Based on Cable‐Driven Actuators

**Figure 8:**
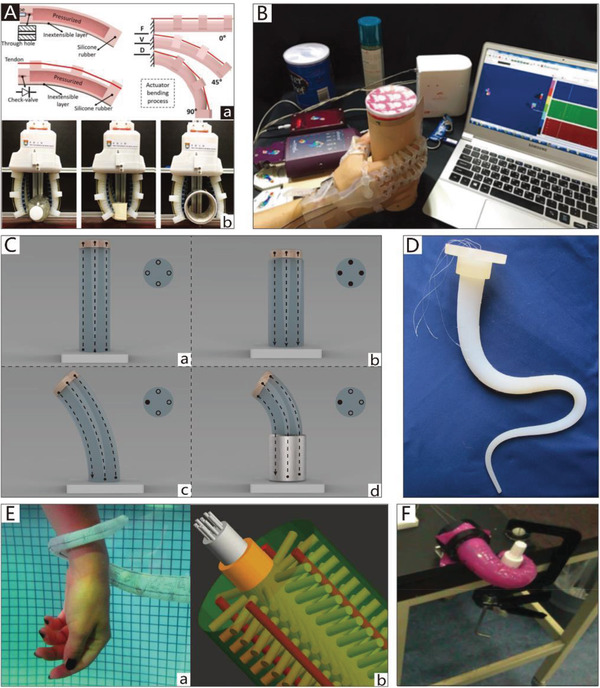
CBGs, CTGs based on cable‐driven actuators. A) A precharged pneumatic soft gripper. Reproduced with permission.^[^ ^56^ ^]^ Copyright 2018, Mary Ann Liebert, Inc. B) A soft wearable robot for hands. Reproduced with permission.^[^ ^57^ ^]^ Copyright 2019, Mary Ann Liebert, Inc. C) The schematic diagram of CTGs based on cable‐driven actuators. The dotted lines show the hidden arrangement of the cables and the circle on the right of each picture represents the cross section of the cylinder in which the filled dots represent pulled cables, and empty dots represent released cables. (Modified and redrawn.) Reproduced with permission.^[^ ^90^ ^]^ Copyright 2011, Elsevier. D) A cable‐driven biomimetic gripper. Reproduced with permission.^[^ ^74^ ^]^ Copyright 2012, IOP Publishing. E) A soft arm inspired by the octopus. Reproduced with permission.^[^ ^75^ ^]^ Copyright 2012, Taylor & Francis. F) A variable compliance, soft gripper. Reproduced with permission.^[^ ^89^ ^]^ Copyright 2014, Springer Nature.

A continuum soft gripper can be considered as a bending manipulator consisting of elastic elements with ideally infinite DOFs.^[^ ^199^ ^]^ The working pattern of cable‐driven actuators is quite suitable for CBGs. Therefore, cable‐driven actuators are also widely applied in the field of CBGs. One example is a pre‐charged pneumatic soft gripper that includes a combination of SPAs and tendon‐driven actuators. The gripper is precisely controlled by the tendons and powered by pre‐charged air. Each finger of the gripper bends to different degrees when the tendons are pulled or released. As depicted in **Figure** **8**A(a), the soft finger has three control parameters, namely the tendon pulling/releasing displacement, the tendon pulling force, and the tendon pulling/releasing speed. One parameter can be selected at a time for control. The soft gripper prototype is shown in Figure 8A(b), and is composed of a gripper base and three fingers that can grasp various subjects, such as a bottle of water, a piece of tofu, and a roll of tape.^[^ ^56^ ^]^ Additionally, Zhu et al.^[^ ^200^ ^]^ proposed a novel spherical self‐adaptive gripper that has a continuous elastic membrane made of a deformable material. The elastic membrane, which is connected to one end of the cable, can envelop and pick up an object with the pulling‐up motion of the cable, and can adapt to objects with various shapes and sizes.

Another application of cable‐driven actuators is their use in soft wearable robots, which help people pinch and grasp. Human hands could be considered as cable‐driven grippers when people equip the wearable robot. Kang et al. developed a series of soft wearable robots for the hands, which are constructed of polymer materials and driven by cable‐driven actuators. The latest soft wearable robot consists of a glove, tendon sheaths, and an actuation system (Figure 8B), and can restore the ability of the hands to pinch and grasp various objects in daily life. Due to the reduced number of components, the robustness of the glove is improved and its weight is decreased, which is critical to the wearing comfort.^[^ ^57^, ^88^ ^]^ The working principle of cable‐driven actuators is the most similar to that of human hands, which will exceptionally meet the needs of soft wearable robots to help spinal cord injury patients accomplish daily tasks.

Like SPAs, cable‐driven actuators have been widely applied in industry. However, they cannot change their stiffness by themselves, as can SPAs. Therefore, they are usually combined with other stiffness‐controllable materials when applied in soft grippers. In the future, cable‐driven actuators will be widely applied in the field of soft wearable equipment due to their excellent characteristics, such as those of human tendons. Due to the particularity of their applications, the materials of soft wearable equipment are usually required to have no toxicity and allow for sanitization. Silicone (KE‐1300T) is a common choice, as it has ability to adapt to different sizes and can be easily cleaned compared with fabric.^[^ ^57^ ^]^ Moreover, to guarantee the mechanical characteristics of the body, sweat volatility and antibacterial activity should be taken into account for wearable equipment in future work.

#### 3.2.4 CTGs Based on Cable‐Driven Actuators

Cable‐driven actuators are characterized by the ability to precisely control their position and force, a high tensile strength in the longitudinal axis direction, and inherent compliance, which make them suitable for the complex motions of CTGs. Many CTGs have been successfully designed on the basis of cable‐driven actuators, the ideally infinite DOFs and hyper‐redundancy of which improve the maneuverability of grippers.^[^ ^201^, ^202^, ^203^ ^]^ The basic working principle of CTGs based on cable‐driven actuators is shown in Figure 8C. The mock‐up, as shown in Figure 8C(a), includes a silicone cylinder and 4 longitudinal cables embedded in the silicone material. When all the cables are pulled, the mock‐up shortens, and the diameter increases (Figure 8C(b)). As shown in Figure 8C(c,d), the mock‐up is driven by pulling a longitudinal cable; in particular, the stiffening of a part of the mock‐up (represented by the rigid tube shown in Figure 8C(d)) can avoid the shortening, elongation, or bending actions of the corresponding part.^[^ ^90^ ^]^

Recently, researchers have drawn inspiration from biology to study CTGs with excellent properties. For example, Laschi et al. developed a series of biomimetic CTGs based on octopus tentacles, which are entirely non‐rigid structures with many DOFs and high flexibility. One of these CTGs is a cable‐driven continuum soft manipulator based on a 3D geometrically‐exact steady‐state model, as illustrated in Figure 8D. The soft manipulator is composed of a single conical piece of silicone driven by low‐stretch cables, which are dispersedly fixed inside the silicone body and coated with a polymer sheath to avoid friction resistance generated between the cables and the silicone body of the manipulator. The general geometrically‐exact steady‐state model of the soft manipulator is so accurate that the model can be easily used to define essential characteristics of motion.^[^ ^74^ ^]^ A supervised learning method and model‐based method have been proposed to solve the inverse statics of the cable‐driven continuum manipulator, and can improve the accuracy and rapidity of the cable‐driven soft manipulator.^[^ ^204^ ^]^

In addition to research on the control methods of biomimetic CTGs, the antagonistic actuation principle of muscular hydrostats (The typical feature of octopus tentacles is their composition of transverse, longitudinal, and obliquely‐orientated muscle groups. The structures formed by the muscular organization are called muscular hydrostats, and their volume is constant during muscle contraction.^[^ ^205^, ^206^ ^]^ Consequently, if the muscular hydrostats extend in the axial direction, their radial dimension will decrease, and vice versa.^[^ ^90^ ^]^) has been applied in a soft bionic gripper by Laschi et al.^[^ ^75^ ^]^ (Figure 8E(a)). The proposed soft biomimetic gripper has some characteristics of the octopus tentacle, which are manifested by imitating the longitudinal and transverse muscles of the octopus. The motion of the longitudinal muscles is achieved by cables, and the motion of the transverse muscles is achieved by SMA springs arranged along the radial direction. The schematic design of the bionic muscular hydrostat unit is shown in Figure 8E(b). It is covered by a support structure that is composed of a braided sleeve and a central channel for electric wires, similar to the nerve cord channel in octopus tentacles.^[^ ^207^ ^]^ Bending motion can be achieved by pulling single or two cables that are considered as longitudinal actuators. Shortening motion can be generated by pulling all the cables simultaneously. SMAs are used to elongate the tentacle or to increase its stiffness when the cables are simultaneously activated and used as antagonistic actuators.

Following the bionic design of octopus tentacles, Laschi et al. developed a soft octopus robot with eight soft arms connected to the base of the robot, which achieve the ability to move and grasp by wrapping around objects. To complete both grabbing and walking motions simultaneously, different types of arms, namely those respectively specialized for manipulation and locomotion, were designed. Two manipulation grippers can implement multiple types of motion, including contracting motion, bending motion, and stiffness variation. They are equipped with motor‐driven cable actuators and SMA springs that are similar to the previous design of Laschi et al.^[^ ^75^ ^]^ The remaining six arms are used for walking, in which a crank mechanism is embedded for the implementation of octopus‐inspired locomotion.^[^ ^208^ ^]^ Each locomotion arm is part of a three‐bar mechanism, which replicates the four phases of the typical pushing action of the octopus arm.^[^ ^209^ ^]^

The ability of the grasping manipulators to accommodate various objects in their surroundings is also essential for cable‐driven grippers. To this end, Giannaccini et al. reported a versatile grasping device driven by inner cables, which can adapt to multiple types of objects due to its entirely soft bionic structure (Figure 8F). This variable‐compliance gripper is inspired by invertebrates with hydrostatic skeletons, whose stiffness can change with the contraction of incompressible fluid‐filled cavities surrounded by muscles.^[^ ^210^ ^]^ The grasping process is divided into two stages. In the first stage, the stiffness of the gripper is so low that the gripper has enough compliance to create a higher contact area with objects. In the second stage, it is necessary to decrease the compliance of the gripper to retain its grasping action.^[^ ^89^ ^]^ Another compliance‐controllable soft manipulator was developed by Stilli et al., which combines the advantages of both cable‐driven and pneumatic actuation mechanisms; accurate position control can be achieved using cables driven by motors, while the pneumatic actuator can be used to implement variable compliance. Compared with a single actuator, the manipulation capabilities of hybrid actuators are generally enhanced.^[^ ^211^ ^]^ The soft manipulator can elongate along its longitudinal axis, bend in all directions based on the principle of antagonistic actuation, and change its compliance by inflating the entire soft outer sleeve module while simultaneously tightening the tendons.^[^ ^212^ ^]^

Compared with other soft grippers based on cable‐driven actuators, CTGs based on cable‐driven actuators have a wider workspace in 3D space, which benefits from the movement form of twisting motions. The generation of twisting motions depends on the cooperation between longitudinal and transverse actuators. These are usually made of soft fibers with high tensile strength, such as UHMWPE synthetic fibers,^[^ ^75^ ^]^ nylon fibers,^[^ ^212^ ^]^ and steel fibers,^[^ ^209^ ^]^ to ensure the accuracy of action. The material of the gripper body also has an impact on twisting motions; soft polymers, such as silicone (ECOFLEX 00–30),^[^ ^204^ ^]^ are often used to ensure the flexibility of the gripper.

Corresponding studies of CTGs based on cable‐driven actuators have been relatively matured, and these actuators are expected to have widespread applications in situations requiring high control precision and low loads, like surgical robots. However, compared with their counterparts in nature, such as octopus tentacles, there is much room for the improvement of their flexibility and response speed. Researching and learning the controlling mechanisms of muscular hydrostats and implementing designs with the concept of modularization could be expected in future work.

### 3.3 Soft Grippers Based on SMAs

#### 3.3.1 Shape Memory Alloys

**Figure 9:**
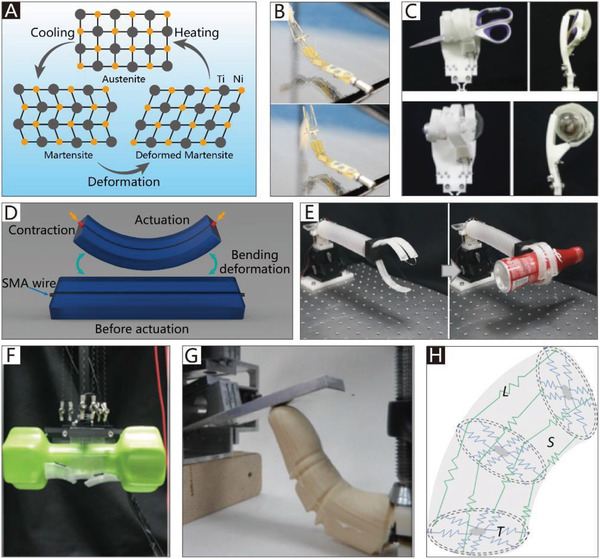
Soft grippers based on SMAs. A) The shape memory effect of NiTi alloys. (Modified and redrawn.) Reproduced with permission.^[^ ^225^ ^]^ Copyright 2018, Springer Nature. B) A wireless folding gripper. Reproduced with permission.^[^ ^51^ ^]^ Copyright 2017, The American Association for the Advancement of Science. C) A soft hand with the excellent bending capacity. Reproduced with permission.^[^ ^52^ ^]^ Copyright 2016, Elsevier. D) Schematic diagram showing the mechanism of the SMA wire‐based soft actuator. (Modified and redrawn.) Reproduced with permission.^[^ ^52^ ^]^ Copyright 2016, Elsevier. E) An SMA‐based soft gripper. Reproduced with permission.^[^ ^60^ ^]^ Copyright 2017, Elsevier. F) A gripper with SMA springs. Reproduced with permission.^[^ ^61^ ^]^ Copyright 2019, IOP Publishing. G) An anthropomorphic finger actuated by the SMA plate. Reproduced with permission.^[^ ^227^ ^]^ Copyright 2015, IOP Publishing. H) A completely soft octopus‐like robotic arm. Reproduced with permission.^[^ ^76^ ^]^ Copyright 2012, IEEE.

SMAs have been developed for many applications,^[^ ^213^, ^214^, ^215^, ^216^, ^217^, ^218^, ^219^, ^220^, ^221^ ^]^ and can be made from alloying zinc, copper, gold, and iron. Copper–aluminum–nickel and nickel–titanium (NiTi) alloys are the most frequently used SMAs. Cu‐based alloys are brittle in a polycrystalline state,^[^ ^222^ ^]^ while NiTi alloys are preferable for most applications due to their stability.^[^ ^223^ ^]^ Due to the shape memory effect of SMAs, they undergo plastic deformation at low temperatures and return to their original configuration after heating.^[^ ^224^ ^]^ This effect is based on the presence of two stable crystalline phases in SMAs, namely the reversible transformation from martensite to austenite, as shown in **Figure** **9**A.^[^ ^225^ ^]^ Martensite is thermodynamically stable at lower temperatures, while austenite is the parent phase that is stable at higher temperatures. Martensite presents a low‐symmetry crystal structure, such as cubic, rhomboid, orthogonal, monoclinic, or triclinic, depending on the composition of the alloy, while austenite usually has a higher symmetry based on a cubic lattice.^[^ ^226^ ^]^

SMAs have the advantages of a high energy density and excellent biocompatibility in operation. In addition, they can be deformed in many different shapes without a substantial loss of actuation performance.^[^ ^94^ ^]^ Moreover, due to the conductivity of SMAs, they can be conveniently driven by Joule heating. However, some drawbacks limit the use of SMAs, namely their lower energy efficiency (1–10%), lower strain rate (<8%), limited bandwidth, and nonlinearity.^[^ ^226^ ^]^

SMAs are widely used in soft grippers covering all application scenarios, including NBGs, CBGs, and CTGs. As shown in Table 2, a high load capacity is an advantage of SMAs; however, a longer cooling time is required for high‐power actuators. Accordingly, making trade‐offs between the load capacity and response time is inevitable when SMAs are used as actuators. Furthermore, compared with soft grippers driven by SPAs or cable‐driven actuators, an advantage of soft grippers driven by SMAs is that it is possible to perform a local bend in a desired portion of the arm without influencing the rest of it, which makes the movement of the gripper more flexible and controllable.^[^ ^94^ ^]^

#### 3.3.2 NBGs Based on SMAs

Different from cable actuators, SMAs used in NBGs usually act as both “muscles” and “tendons.” More concretely, one end of the SMA wire is fixed in the base of the gripper that does not move, and the other end is attached to the phalanx that can rotate around the joint. SMA wire can produce a forceful contraction like the skeletal muscle of humans. Based on this method, Simone et al.^[^ ^228^ ^]^ reported a bio‐inspired hand‐like gripper, each finger of which is mounted with two different bundles of SMA wires prepared for different joints. Because the SMA wires can directly transmit motion to each hinge, various achievable gripper configurations can be implemented, some of which are difficult for human hands and NBGs based on cable‐driven actuators.

Generally, SMAs produce a driving force via Joule heating, which requires a battery or external power supply with wires. Due to the low efficiency of SMAs (1–10%), the battery does not guarantee long‐term operation,^[^ ^229^ ^]^ and an external power supply with wires is also not ideal because the wires could limit the movement range of the gripper. To address this issue, Boyvat et al.^[^ ^51^ ^]^ reported an untethered folding gripper based on self‐folding laminate structures with flexure hinges, which exploits wireless electromagnetic power transmission. The untethered folding gripper has three LC resonances that generate induced current by an external magnetic field. Due to the selective excitation of the LC resonances, SMA actuators can be individually or collectively activated. It can be seen from Figure 9B that the untethered folding gripper can pick up a sponge, hang it in the air, and then drop it.

A broad range of motion is another challenge of NBGs based on SMAs. To break through this bottleneck, Kim et al.^[^ ^52^ ^]^ designed a soft hand with a good bending capacity (a bending angle of 305° with a weight of 20 g). The tendon‐driven artificial finger of the soft hand has a resemblance to the human finger. It has three soft hinges that amplify the deformation of the SMA wire by an SMA sliding mechanism; the soft hinges are more compliant than the other regions of the fingers, which allows the SMA wire to achieve massive displacement. Furthermore, the two tendon‐driven wires are similar to human finger tendons. Therefore, the finger can achieve similar bending behavior as the human finger. As illustrated in Figure 9C, the soft hand can be used to grab objects, such as a pair of scissors and a light bulb, with different fingers.

NBGs based on SMAs are usually lightweight and have a tight configuration, which is attributed to their high energy density and smaller diameter (as low as 100 µm^[^ ^228^ ^]^). However, their response speed is limited, and partially depends on the cooling rate. Although there exist many methods by which to decrease the response time, such as reducing the diameter of the SMA wires or adding additional cooling equipment, the former may adversely influence the power, and the latter requires extra energy consumption. Furthermore, their relatively low holding force (a load‐bearing capability of 60 g^[^ ^52^ ^]^) is another reason why NBGs based on SMAs are seldom adopted in practice.

#### 3.3.3 CBGs Based on SMAs

Due to the advantages of SMAs mentioned at the beginning of this section, they have been widely applied in CBGs. The basic bending mechanism of CBGs based on SMAs is shown in Figure 9D. When the SMA contracts via heating, the actuator exhibits bending and twisting deformation due to the eccentricity of the SMAs. The movement of the actuator can be changed by varying the position of the wires.^[^ ^52^ ^]^

Figure 9E presents an example of SMA‐based soft grippers with initial curvature. The gripper is composed of three curved fingers, and its maximum grasping force is about 1.5 N. Each finger is an executive component driven by an embedded SMA wire. Moreover, the maximum bending angle of the finger can be accurately predicted according to the initial bending angle.^[^ ^60^ ^]^ She et al.^[^ ^93^ ^]^ presented a soft robotic hand with a pinching force of about 3 N. The bending actuators of the hand are SMA strips, and the proposed analysis model can precisely predict the output performances.

As mentioned previously, SMAs can maintain consistent actuation performance for many different shapes, and can be driven by Joule heating without additional devices. These advantages make SMAs suitable for miniaturization. For instance, Lan et al. designed a compliant SMA‐based microgripper with two 1‐cm arms made of SMAs and polyoxymethylene, which can achieve a large gripping range and force in a limited space. The microgripper can even manipulate a string with a diameter of 0.1 mm.^[^ ^92^ ^]^

SMA‐based soft grippers are usually driven by Joule heating, and the problem of dissipating heat is an essential factor that confines the recovery speed of SMAs. To increase the recovery speed of SMAs, Lee et al.^[^ ^61^ ^]^ developed a gripper with SMA springs as external tendons to improve the grasping performance (Figure 9F). Positioning the SMA springs externally has two advantages. First, the open environment assists in heat dispersion, and the implementation of active cooling via fans can improve the response performance of the SMA. Second, the external positioning of the SMA springs allows for more space to arrange larger SMA springs, which would allow for the increase of power. The gripper has the capacity to hold a weight of 2 kg (with pull‐up forces of up to 30 N). It can also accomplish cyclic actuation at 0.25 Hz based on proper cooling. Placing the gripper into cold water is another cooling method, as shown in Figure 9G (the force and displacement of the finger are measured). This anthropomorphic finger is actuated by an SMA plate, which can take the shape of a flexed human finger when the SMA is joule‐heated. The finger was found to exhibit low tracking errors in square‐wave and sinusoidal tracking experiments when it was wholly submerged underwater, as the cooling effect can improve the response of the SMA. The maximum force of the fingertip is 9.01 N on average when the actuator material of the SMA plate is composed of Ni_50.1_Ti_49.9_.^[^ ^227^ ^]^

#### 3.3.4 CTGs Based on SMAs

Compared with SPAs and cable‐driven actuators, SMAs are not the best choice for longitudinal actuators. Their response time and deformability (strain rate < 8%) are not ideal, and their energy consumption is high.

There is an exception that is a gripper based on longitudinal and transverse actuation units, which are composed of SMA springs. Compared to CTGs based on SPAs and cable‐driven actuators, one of the advantages of longitudinal SMA actuators is that it is possible to perform a local bend in a desired portion of the arm without influencing the rest of the actuator. Furthermore, to overcome the defect of the lower strain rate, SMAs have been shaped into coils, thereby yielding spring‐like actuators that can produce more significant displacements.^[^ ^94^ ^]^ Similarly, another functional unit of a completely soft octopus‐like robotic arm was developed by Laschi et al.^[^ ^76^ ^]^ The functional unit is composed of soft actuators and a support structure, as shown in Figure 9H. SMA springs are chosen for the actuators (longitudinal actuators (L) and transverse actuators (T)), and the support structure (S) is a braided sleeve that provides spatial continuity for the action of the SMA springs. The functional unit can achieve elongation, shortening, and bending driven by actuators with different control strategies. In addition, Zhang et al.^[^ ^230^ ^]^ reported a spatial soft module actuated by three SMA coils that can implement multiple types of bending motion.

### 3.4 Soft Grippers Based on SMPs

#### 3.4.1 Shape Memory Polymers

**Figure 10:**
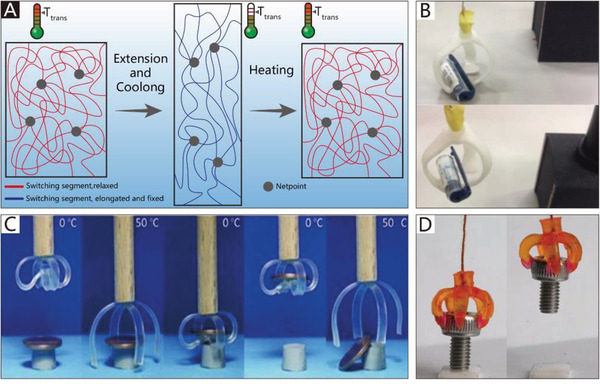
Soft grippers based on SMPs. A) The process of deforming and recovery of an SMP. (Modified and redrawn.) Reproduced with permission.^[^ ^236^ ^]^ Copyright 2002, John Wiley and Sons. B) A gripper is driven by SMPs. Reproduced with permission.^[^ ^58^ ^]^ Copyright 2015, Springer Nature. C) A bidirectional gripper based on SMPs. Reproduced with permission.^[^ ^59^ ^]^ Copyright 2013, John Wiley and Sons. D) A 3D printed SMP gripper. Reproduced with permission.^[^ ^96^ ^]^ Copyright 2016, Springer Nature.

SMPs are stimuli‐responsive materials and have been widely used in various research fields.^[^ ^231^, ^232^, ^233^, ^234^, ^235^ ^]^ They can rapidly change their shape when heated or exposed to other external stimuli. **Figure** **10**A shows the deformation and recovery process of SMPs,^[^ ^236^ ^]^ from which it can be seen that an SMP sample is in a permanent shape at the beginning, and after being heated, deformed, and cooled, the sample then changes to a temporary shape. Finally, the sample is heated at a temperature above the transition temperature to induce the shape memory effect; thus, the permanent shape is obtained.^[^ ^237^ ^]^ The elastic deformation of SMPs can reach 200%, and they have the advantages of a lower density, easy processing, and potential biocompatibility.^[^ ^238^ ^]^

Nevertheless, due to the low recovery stress of SMPs (only 1–3 MPa,^[^ ^239^ ^]^ which is far below that of SMAs, namely 150–300 MPa^[^ ^238^, ^240^ ^]^), grippers driven by SMPs are only suitable for situations involving low loads. Moreover, unlike SMAs, which can be driven directly by Joule heating, SMPs usually require an external heater, which could impose an extra load on the gripper. In fact, compared with being used as an actuator, the use of SMPs as variable stiffness components in combination with other actuators is the main application of SMPs, which is discussed in Section 4.4.

#### 3.4.2 CBGs Based on SMPs

Due to the deformation and recovery stresses of SMPs, which are both only 1–3 MPa,^[^ ^239^ ^]^ they are only suitable for situations involving low loads. Moreover, compared with other soft actuators, their recovery speed, which is only 1 s to several minutes,^[^ ^239^ ^]^ is not dominant. Thus, SMPs are rarely used as actuators in soft grippers.

One exception is a gripper developed by Yang et al.,^[^ ^58^ ^]^ which can be manufactured by fused deposition modeling. According to the thermal sensitivity of SMPs, the gripper can pick up a pen cap when it is heated above the glass transition temperature, as shown in Figure 10B. Another study based on reversible, bidirectional SMP is reported by Behl et al.,^[^ ^59^ ^]^ which is assembled by polymer ribbon made from PPD‐PCL(75). As illustrated in Figure 10C, the gripper can reversibly grasp and release a penny by heating or cooling. Furthermore, some advanced processing techniques, like the 3D printing approach, can also be used to manufacture CBGs based on SMPs. One example is shown in Figure 10D, after triggered upon heating, the gripper can pick up a screw.^[^ ^96^ ^]^

### 3.5 Soft Grippers Based on DEAs

#### 3.5.1 Dielectric Elastomer Actuators

Electroactive polymers (EAPs) are characterized by the advantages of a low weight, rapid response, and relatively large actuation strain, which are suitable for soft actuators and robots.^[^ ^241^, ^242^, ^243^, ^244^ ^]^ EAPs are broadly categorized into two major groups based on their driving principle. The first is electronic EAPs, which are driven by electric fields or Coulomb forces. Their typical representatives are dielectric elastomers and LCEs. The second group is ionic EAPs, which are driven by the mobility or the diffusion of ions. Their typical representative is IPMCs.^[^ ^245^ ^]^

**Figure 11:**
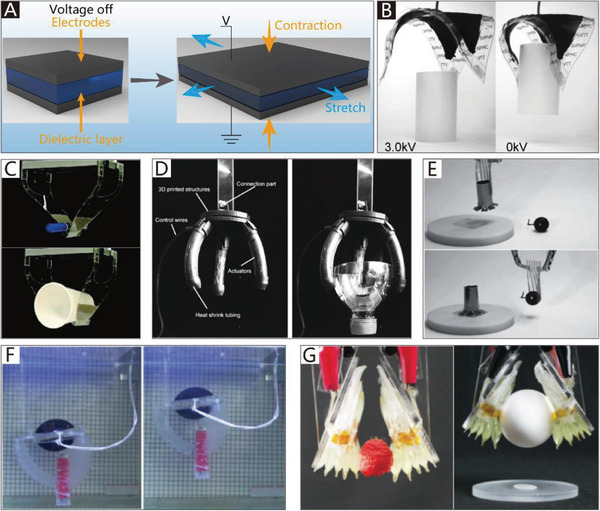
CBGs based on DEAs. A) The working principle of DEAs. (Modified and redrawn.) Reproduced with permission.^[^ ^247^ ^]^ Copyright 2018, MDPI AG. B) A soft gripper based on DEME Reproduced with permission.^[^ ^62^ ^]^ Copyright 2007, AIP Publishing. C) A responsive DEME soft gripper. Reproduced with permission.^[^ ^248^ ^]^ Copyright 2019, Springer Nature. D) A soft gripper with spring‐roll bending actuators. Reproduced with permission.^[^ ^249^ ^]^ Copyright 2019, Mary Ann Liebert, Inc. E) A light and flexible DEA gripper. Reproduced with permission.^[^ ^250^ ^]^ Copyright 2015, John Wiley and Sons. F) A hydraulic gripper driven by the DEME actuator. Reproduced with permission.^[^ ^251^ ^]^ Copyright 2018, IOP Publishing. G) An electrostatic gripper based on hydraulically amplified mechanism. Reproduced with permission.^[^ ^63^ ^]^ Copyright 2018, The American Association for the Advancement of Science.

In this section, the use of dielectric elastomers (DEs) as a motion‐generating material is discussed, as DEs resemble human skeletal muscles in terms of both strength and tension.^[^ ^246^ ^]^ DEAs are usually composed of two compliant electrodes and a DE film sandwiched between them. When a high voltage is applied, electrostatic forces are generated by the Coulomb forces acting between the compliant electrodes. Thus, the DE film is compressed along the axial direction and stretched along the radial direction of the film. The working principle is depicted in **Figure** **11**A.^[^ ^247^ ^]^

The advantages of DEAs are that they are soft, compliant, lightweight, and have a fast response speed (over 2000 Hz),^[^ ^252^ ^]^ high efficiency (80%),^[^ ^253^ ^]^ large actuation strain (more than 300%),^[^ ^254^, ^255^ ^]^ and a considerable actuation stroke (an expansion of area up to 1692%).^[^ ^256^ ^]^ Besides, silicone and acrylic elastomers also have high energy densities of about 10 and 150 kJ m^−3^, respectively, compared to the energy density of 40 kJ m^−3^ in mammalian skeletal muscle.^[^ ^257^ ^]^ Therefore, they are suitable for driving soft bionic grippers. However, the most significant disadvantages of DEAs are their high voltage requirement (150 MV m^−1^), which is considered unsafe, and their short service life due to degradation during use.^[^ ^258^ ^]^

#### 3.5.2 CBGs Based on DEAs

Dielectric elastomer minimum energy structures (DEMES) are typical structures of DEAs used in soft grippers, which usually consist of pre‐stretched DEA membranes and flexible, but not stretchable, frames.^[^ ^241^, ^242^ ^]^ In other words, they are created by combining energy and entropy elasticity in bending frames and stretched elastomers. Due to the internal stress of the pre‐stretched DEA, the actuator is in a passive bent state. When applying a voltage to the DEMES, the DEA membrane becomes thinner, and its internal stress is released. Consequently, the frame of the actuator pushes the actuator to open.^[^ ^259^, ^260^ ^]^ A soft gripper based on DEMES reported by Kofod et al.,^[^ ^62^ ^]^ which is composed of a pre‐stretched elastomer and a triangular frame, is illustrated in Figure 11B. The soft gripper opens when a voltage of 3.0 kV is applied. After the removal of voltage, the soft gripper can close around and pick up an object. Figure 11C presents another DEMES soft gripper with a quick response speed, which can completely open or close in 0.179 s. The soft gripper consists of two bi‐stable‐structure actuators with two layers of pre‐stretched DEAs and an inextensible flexible PET sheet, which is inspired by the bi‐stable behavior of the Venus flytrap.^[^ ^248^ ^]^

The gripping force of grippers based on DEMES depends on the elastic potential energy of the flexible frame, which is usually composed of an elastic sheet. The conventional materials for elastic sheets include transparent polyester foil,^[^ ^261^ ^]^ a plastic film frame,^[^ ^259^ ^]^ and polyethylene terephthalate,^[^ ^248^ ^]^ the stiffnesses of which are relatively low, which usually results in a low holding force. An example is a gripper based on DEAs with a maximum gripping force of 2.2 mN.^[^ ^261^ ^]^ These factors could hinder the application of DEMES in CBGs.

To improve the load‐holding capacity of CBGs based on DEAs, Li et al.^[^ ^249^ ^]^ produced a soft gripper using a pre‐compressed spring instead of the traditional elastic sheet as a flexible frame (Figure 11D). The gripper is assembled with three spring‐roll bending actuators that consist of a pre‐compressed spring and biaxial pre‐stretched VHB film with carbon grease electrodes. The electrodes at one side of the actuator drive the VHB film to produce asymmetric deformation. When a suitable voltage (from 2 to 6 kV) is applied, one side of the VHB film is released, while the other side remains unchanged. Therefore, the pre‐compressed spring bends toward the side at which the VHB film is released. The measured pull‐out force is 228.3 mN when the applied voltage is 5 kV. However, the actuator will fail (dielectric breakdown) when a too‐high voltage, such as 5.5 kV, is applied for a long time.

Previous grippers used pre‐stretched DEAs that require relatively stiff and non‐stretchable frames to maintain structural integrity. The existence of these frames will increase the weight of the grippers and reduce their overall flexibility.^[^ ^99^ ^]^ To endow soft grippers with a lighter weight, Shian et al.^[^ ^250^ ^]^ designed a flexible DEA gripper that incorporates a few stiff fibers to alter the shaping functionality in response to voltage. Different from DEMES, the DEA membrane of this gripper does not need to be pre‐stretched, and a flexible frame is not required. The few stiff fibers can break the original symmetry of the DEA gripper to induce deformation and predetermine the direction of shape changes. As shown in Figure 11E, the gripper can adapt to different objects, such as a cylinder and a grape.

In the examples discussed previously, the grippers are directly driven by DEAs. Due to the insecurity generated by a high voltage, the DEAs are not suitable as executive devices for direct contact with the manipulated objects. However, the use of DEAs as drivers could provide a safe option; based on this concept, Zhang et al.^[^ ^251^ ^]^ proposed a novel driving system that integrates a DEA into a hydraulic gripper, as illustrated in Figure 11F. The DEA is used as a soft hydraulic source, which is assembled with an acrylic frame and a dielectric elastomer balloon that consists of a carbon grease electrode and two biaxially pre‐stretched dielectric elastomer membranes. The soft robotic gripper is driven by hydraulic pressure from the DEA actuator, rather than directly by the DEA actuator. The hydrogel chamber, as the execution unit, is connected to the DEA by a tube. The entire device is pumped with water before working. When a high voltage is applied, the dielectric elastomer balloon expands, and the inner pressure of the device decreases, leading to the variation of the bending angle of the gripper.

As an important component of DEAs, DE films have the advantages of a low stiffness, high dielectric constant, and high electrical breakdown strength, and typical materials include acrylic elastomers, silicones, polyurethanes, and rubber. Acrylic elastomers, represented by VHB4910, enjoy the widest application because of their low stiffness, which translates to larger deformations at the same pressure. DE films based on silicones, represented by PDMS, and polyurethanes have a faster response speed, but their strain is relatively less. Rubber, compared with VHB film, has a higher breakdown strength that is attributed to its higher Young's modulus (up to ten times higher than that of VHB film).^[^ ^262^, ^263^ ^]^

DEAs are driven by high voltage. The failure of DE films is usually caused by dielectric breakdown and electrical aging.^[^ ^264^ ^]^ To solve the failure problem, Acome et al.^[^ ^63^ ^]^ reported a hydraulically amplified self‐healing electrostatic gripper with robust, muscle‐like performance. The actuator of the gripper is a soft‐shell filled with a liquid dielectric elastomeric and covered by a pair of opposing electrodes. Compared to traditional DEAs, hydraulically amplified self‐healing electrostatic actuators have the ability to scale the actuation force and strain. In contrast to solid dielectrics, such as VHB films, which become permanently damaged from dielectric breakdown, liquid dielectrics can immediately return to an insulating state and self‐heal from electrical damage. When a suitable voltage is applied to the actuators, the device can grasp a raspberry or a raw egg, as shown in Figure 11G. Moreover, a gripper combined with six planar hydraulically amplified self‐healing electrostatic actuators can lift a gallon of water (≈4 kg), which demonstrates its potential power under heavy‐load working conditions. Using similar principles, Park et al.^[^ ^265^ ^]^ developed a soft gripper driven by an electrohydraulic actuator, which can be used to grab delicate materials.

The greatest obstacle for the practical application of DEAs is their safety and service life. The ultrahigh driving voltage not only easily causes the dielectric breakdown of DEAs, but also threatens the security of operators. Thus, decreasing the driving voltage to a safe range while ensuring the output power is the wave of the future. Furthermore, most DEAs can only implement planar bending motions, which provide an ample development space in the field of twisting‐type grippers.

### 3.6 Soft Grippers Based on IPMCs

#### 3.6.1 Ionic Polymer Metal Composites

**Figure 12:**
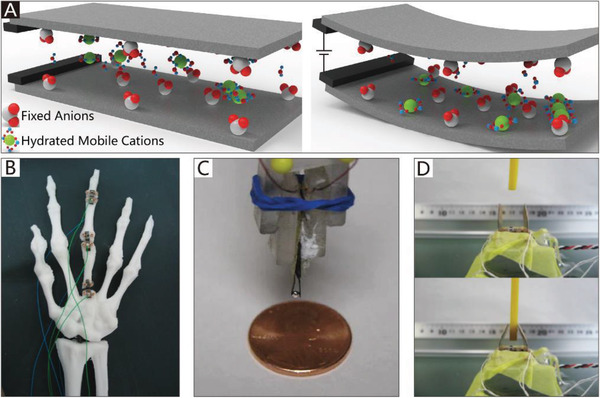
CBGs based on IPMCs. A) The working principle of IPMCs. (Modified and redrawn.) Reproduced with permission.^[^ ^268^ ^]^ Copyright 2014, Mary Ann Liebert, Inc. B) A bionic finger driven by IPMC actuators. Reproduced with permission.^[^ ^53^ ^]^ Copyright 2006, IOP Publishing. C) A microgripper driven by IPMC. Reproduced with permission.^[^ ^101^ ^]^ Copyright 2008,Springer Nature. D) A Venus flytrap‑inspired microrobot consisted of two IPMC actuators. Reproduced with permission.^[^ ^65^ ^]^ Copyright 2015, Springer Nature.

IPMCs, an important type of ionic electroactive polymer,^[^ ^266^ ^]^ consist of a solvent swollen ion‐exchange polymer membrane coated with a thin, flexible layer of metallic particles (typically percolated platinum or gold nanoparticles).^[^ ^267^ ^]^ When a low driving voltage (1–5 V), which is relatively lower compared to that of DEAs, is applied, the hydrophilic positive ions or cations in the membrane can migrate to the cathode,^[^ ^268^ ^]^ as depicted in **Figure** **12**A, which causes one side of the membrane to swell while the other side experiences contraction. This will result in bending actuation.^[^ ^269^ ^]^

IPMCs have applied in many fingered grippers,^[^ ^270^, ^271^, ^272^ ^]^ and exhibit electroactive behavior in both dry and wet environments.^[^ ^273^ ^]^ Among all the available actuators for soft grippers, IPMCs are the suitable ones due to their large bending displacement at a low applied voltage.^[^ ^267^, ^274^ ^]^ Moreover, they have a higher force density, which means that they can lift an object 40 times their own weight.^[^ ^275^ ^]^ However, due to their low output stress, they can only be applied in situations involving a light load. Furthermore, due to the particularity of the driving form, it is difficult for them to generate twisting movements in 3D space; thus, in the field of CTGs, IPMCs are used sparingly.

#### 3.6.2 NBGs Based on IPMCs

Compared with SPAs, cable‐driven actuators, and SMAs, IPMC films have the advantage of being lightweight, and they can produce sizeable bending deformation under a low driving voltage. Therefore, they are a better choice for some specific NBGs.

IPMC films used in NBGs are usually attached between two rigid frames, the relative rotation of which is directly driven by the IPMC films. However, the actuating force of IPMC films is low, which limits their application in larger soft grippers, especially when NBGs are applied to anthropomorphic robots. Bhattacharya et al. designed a soft robotic finger for gripping, which consists of a PDMS frame and three IPMC actuators placed in three joint locations of the finger.^[^ ^100^ ^]^ Another example is shown in Figure 12B, which depicts a bionic finger that uses IPMC actuators as the ligaments of the joints and polyvinyl chloride (PVC) as the bones. To increase the actuating force, a five‐film stacked IPMC actuator is used. When a voltage is applied to the IPMC, the bionic finger is driven. The maximum tip force is acquired by applying a 4 V DC voltage to the IPMC actuator.^[^ ^53^ ^]^

#### 3.6.3 CBGs Based on IPMCs

Compared with DEAs, IPMCs are usually considered to be safe due to their low driving voltages (1–5 V). They have been widely used in soft robots, which are attributed to their bending actuation nature and advantages mentioned previously. However, due to their low output stress, they can only be used under the working condition of a light load. An example is a 4‐finger gripper based on IPMCs, which can lift a mass of 10.3 g.^[^ ^102^ ^]^

Manipulating micro‐objects is one application of IPMC‐based CBGs. Figure 12C presents an example, namely a microgripper developed by Deole et al.^[^ ^101^, ^276^ ^]^ that uses IPMCs as an actuator. The microgripper can hold a solder ball of 15 mg when a force of 85 µN is exerted. In addition to solder balls, flexible objects, such as hydrogel crystals, can also be grasped. To reduce both energy and consumption, Ford et al.^[^ ^64^ ^]^ reported a single‐active‐finger IPMC microgripper that has one stationary finger and one actuating finger. The performance of this microgripper is nearly equivalent to that of a two‐fingered IPMC gripper, and because it uses half of the IPMCs, it is the economical choice. Moreover, Shi et al.^[^ ^65^ ^]^ reported a Venus flytrap‐inspired microrobot consisting of two IPMC actuators and a proximity sensor, which mimics the biological functions of trigger hairs to detect an object moving between the two IPMC actuators. After a series of tests, the maximum grasping force of the IPMC actuators was found to be 36 mN at a voltage of 7 V. As shown in Figure 12D, they can quickly bend toward each other when a plastic stick moves close to the proximity sensor.

### 3.7 Soft Grippers Based on LCEs

#### 3.7.1 Liquid‐Crystal Elastomers

**Figure 13:**
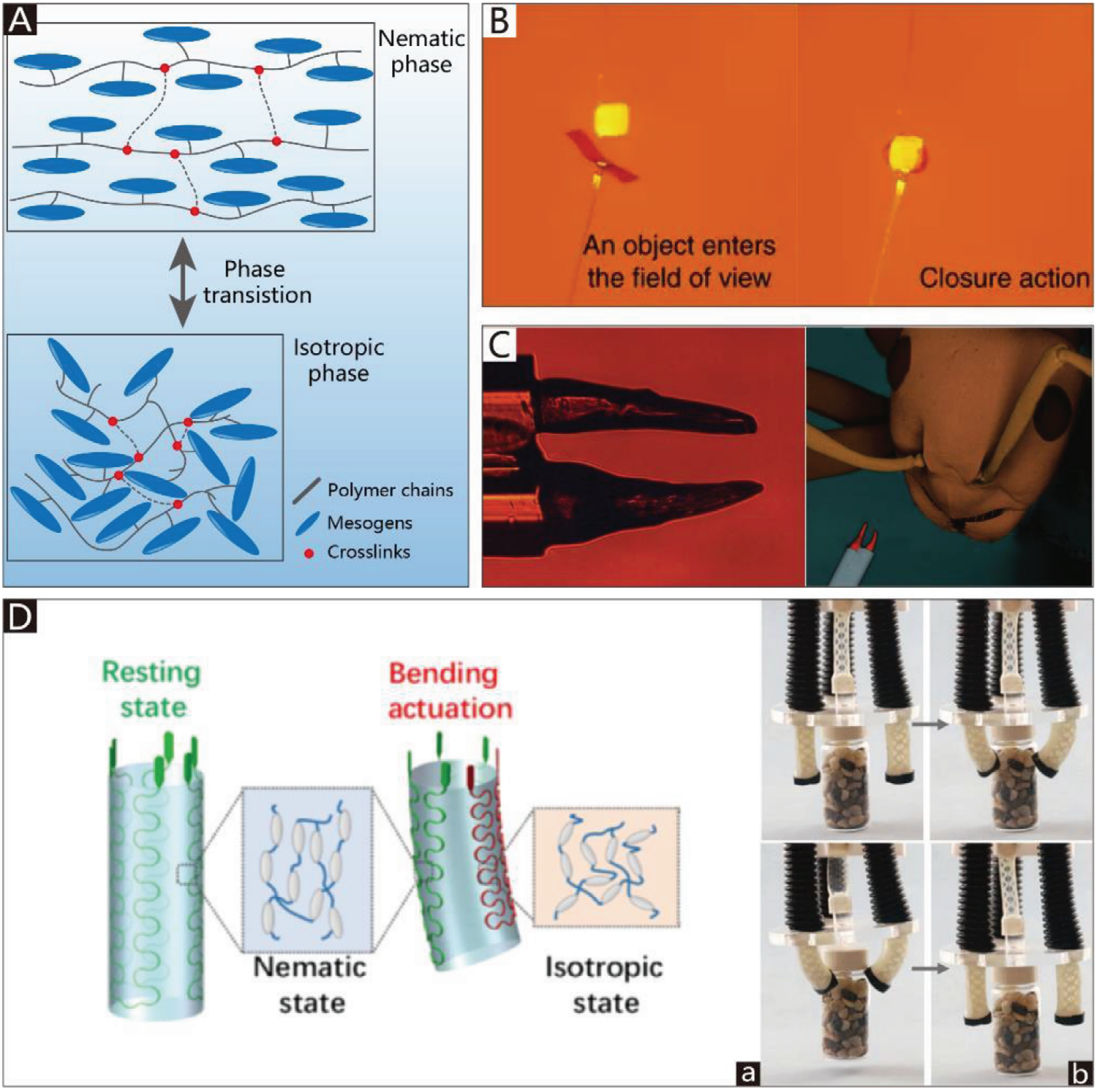
CBGS based on LCEs. A) The working principle of LCEs. (Modified and redrawn.) Reproduced with permission.^[^ ^262^ ^]^ Copyright 2016, John Wiley and Sons. B) A light‐driven artificial flytrap. Reproduced with permission.^[^ ^103^ ^]^ Copyright 2017, Springer Nature. C) A micrometer‐scale, light‐driven plier. Reproduced with permission.^[^ ^66^ ^]^ Copyright 2020, John Wiley and Sons. D) An electrically controlled soft gripper with three LCE tubular actuators. Reproduced with permission.^[^ ^67^ ^]^ Copyright 2019, The American Association for the Advancement of Science.

LCEs are slightly cross‐linked liquid crystalline polymer network materials, and can be used as artificial muscles and in biomedical devices.^[^ ^277^, ^278^, ^279^, ^280^, ^281^ ^]^ They present a combination of the anisotropic features of liquid crystal phases and the elasticity of polymer networks.^[^ ^282^ ^]^ This combination can endow materials with unique properties, such as thermal actuation, anisotropic swelling, and soft elasticity.^[^ ^283^ ^]^ The mesogens in LCEs may be part of the polymer chain (main‐chain liquid crystalline elastomers) or attached via an alkyl spacer (side‐chain liquid crystalline elastomers).^[^ ^284^ ^]^ LCEs have the ability to reversibly change their shape after exposure to certain external stimuli, including temperature, light irradiation, and electric fields. For example, nematic‐phase LCEs, in which the mesogens are randomly aligned and bundled, can convert to an isotropic phase under the action of heating.^[^ ^262^ ^]^ The process can produce reversible and repeatable contractions, as shown in **Figure** **13**A.

#### 3.7.2 CBGs Based on LCEs

LCEs can undergo massive shape changes (≈40%) in response to various external stimuli, such as temperature and light irradiation.^[^ ^285^, ^286^ ^]^ Moreover, their response speed is rapid (on the order of milliseconds); thus, they are an ideal actuator material for CBGs.

One example is a light‐driven artificial flytrap, as shown in Figure 13B, which can mimic the behaviors of a Venus flytrap, such as autonomous closure and object recognition. It can close within 200 ms, which is very similar to the closing time of a natural flytrap (100 ms). The gripping force of the artificial flytrap stems from a light‐responsive LCE actuator that is mounted on the tip of an optical fiber. The light‐driven artificial flytrap can exploit optical feedback to trigger stimulus‐responsive actuation.^[^ ^103^ ^]^ Lahikainen et al.^[^ ^287^ ^]^ took advantage of photothermal responses in light‐active liquid crystal polymer networks to develop another reconfigurable microgripper, which utilizes azobenzene photoisomerization to locally control the cis‐isomer content and program the actuator response. Xiao et al.^[^ ^104^ ^]^ reported another gripper based on liquid crystal network (LCN) actuators that are fabricated by sandwiching a layer of heating wires between a Kapton film and LCN film. The LCN layer serves as the active layer, while the thermostable Kapton film acts as the passive layer. Joule heating can induce the contraction of the LCN and thus trigger the deformation of the actuator. The gripper can grasp an object weighing 7.4 g, which is up to 210 times heavier than the weight of the actuator (34.9 mg).

Besides, in some application scenarios, like gripping and handling sub‐millimeter electronic components or single‐micrometer living cells, the application of traditional grippers is greatly restricted by their complexity and need for force transmission. The use of LCE microactuators that can quickly and reversibly change shape in response to light is one of the solutions to this problem. One example is an optical plier (Figure 13C) made of two bending LCE microactuators (the length of which is equal to about 0.3 mm). The fibers, which are used to send light, are glued together after the fabrication of the LCE microactuators. The LCE microactuators bend in response to the light delivered through the optical fibers and achieve a grasping action.^[^ ^66^ ^]^

#### 3.7.3 CTGs Based on LCEs

The twisting motion of CTGs driven by LCEs can be realized via the asymmetric arrangement of actuators. One example was fabricated with a cross angle of 45° between the alignment directions of two different layers of LCEs, which can execute a right‐handed helical twisting movement like that of a plant tendril when it is irradiated by near‐infrared (NIR) light.^[^ ^105^ ^]^

Thermal stimulation is another way to drive LCE actuators. One example is an electrically controlled soft gripper with three LCE tubular actuators, which sandwich a layer of stretchable heating wires between two layers of loosely cross‐linked LCE films.^[^ ^67^ ^]^ The bending motion of the gripper can be realized by applying an electrical potential to specific heating wires, via Joule heating, one side of the LCE tubular actuator homogeneously contracts, as depicted in Figure 13D(a). The soft gripper based on LCE tubular actuators can grasp and lift a 50 g vial, as shown in Figure 13D(b).

### 3.8 Soft Grippers Based on Other Smart Materials

Stimuli‐responsive smart materials are usually developed to respond to mostly external stimuli, including optical,^[^ ^288^, ^289^ ^]^ thermal,^[^ ^68^, ^290^ ^]^ magnetic,^[^ ^291^, ^292^ ^]^ electrical,^[^ ^293^ ^]^ humidity,^[^ ^294^ ^]^ and chemical (pH change,^[^ ^69^, ^295^ ^]^ salinity^[^ ^296^ ^]^) stimuli. Compared with the quite mature actuators used in soft grippers, such as SPAs and cable‐driven actuators, soft grippers based on smart materials can directly respond to various stimuli and then implement grasping without any force transmission device, external sensor, or extra energy; this allows them to be smaller, so they are more suitable for the manipulation of small‐scale objects. However, most of the existing soft grippers based on smart materials are still in the laboratory research phase. Limited by their driving capability, they can only be operated in situations in which the load demands are minuscule.

#### 3.8.1 CBGs Based on Other Smart Materials

**Figure 14:**
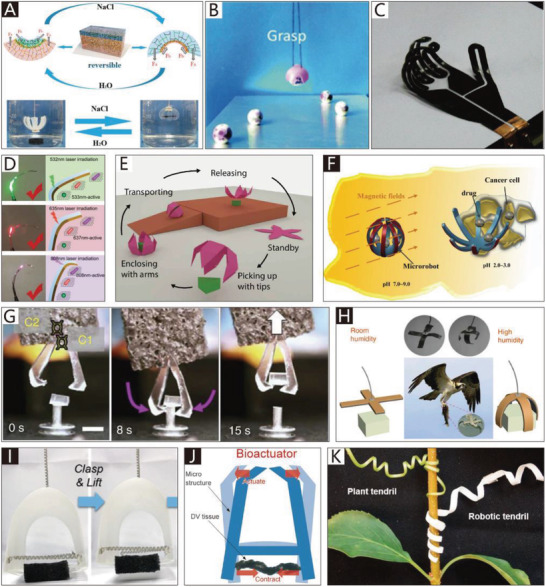
Soft grippers based on other smart materials. A) A salt‐responsive bilayer hydrogel. Reproduced with permission.^[^ ^296^ ^]^ Copyright 2017, American Chemical Society. B) A mimosa inspired bilayer hydrogel gripper. Reproduced with permission.^[^ ^299^ ^]^ Copyright 2018, Royal Society of Chemistry. C) A polymer electrothermal hand. Reproduced with permission.^[^ ^68^ ^]^ Copyright 2015, American Chemical Society. D) A light‐sensitive soft finger. Reproduced with permission.^[^ ^289^ ^]^ Copyright 2018, John Wiley and Sons. E) A programmable micrometer‐scale robot. Reproduced with permission.^[^ ^292^ ^]^ Copyright 2019, The American Association for the Advancement of Science. F) A pH‐responsive hydrogel‐based soft micro‐robot. Reproduced with permission.^[^ ^69^ ^]^ Copyright 2016, IOP Publishing. G) A small‐scale soft gripper. Reproduced with permission.^[^ ^293^ ^]^ Copyright 2013, Springer Nature. H) A soft gripper responded to humidity. Reproduced with permission.^[^ ^294^ ^]^ Copyright 2019, Springer Nature. I) An electrothermal soft gripper. Reproduced with permission.^[^ ^290^ ^]^ Copyright 2018, American Chemical Society. J) An insect muscle powered bio‐actuator. Reproduced with permission.^[^ ^300^ ^]^ Copyright 2016, Mary Ann Liebert, Inc. K) A soft tendril‐inspired twining‐type gripper. Reproduced with permission.^[^ ^77^ ^]^ Copyright 2018, American Chemical Society.

As a common smart material, hydrogels are often used as soft actuators and have shown promising applications in the soft robotics field. Hydrogels are polymers with hydrophilic 3D polymeric networks composed of polymeric chains joined by connecting points or joints.^[^ ^297^ ^]^ Synthesized gels can swell in response to a variety of stimuli by integrating specific molecular moieties into pendant groups or the backbone of the network.^[^ ^298^ ^]^ One example is shown in **Figure** **14**A, which presents a salt‐responsive bilayer hydrogel that includes a polycationic (polyMETAC/HEAA) layer with a polyelectrolyte effect and a polyzwitterionic (polyVBIPS) layer with an anti‐polyelectrolyte effect. Both the polycationic layer and polyzwitterionic layer can respond to changes in solution salinity. However, their response mechanisms are completely contrary. When the polycationic layer displays a swelling behavior, the polyzwitterionic layer displays a shrinking behavior, and vice versa.^[^ ^296^ ^]^ By switching the solution between NaCl and water, the salt‐responsive bilayer hydrogel gripper can bend downward to pick up an object and bend upward to release it.

Responsive hydrogels generally have weak mechanical properties, and one of the solutions to this is the combination of chemical and physical cross‐linking.^[^ ^301^ ^]^ Moreover, the problem of the slow response time of hydrogels remains, which could be solved using porous hydrogels.^[^ ^302^ ^]^ Responsive hydrogels are also highly dependent on aqueous solutions, which limits their performance under air conditions. In air conditions, the expansion volume of responsive hydrogels is much less and the response time is longer than in water conditions.^[^ ^303^ ^]^ To solve this problem, Zheng et al. presented a mimosa‐inspired bilayer hydrogel gripper (Figure 14B), which can generate motions in open‐air environments. The hydrogel gripper is composed of a hydrogel layer derived from a polymer featuring a lower critical solution temperature. The other hydrogel layer is derived from a polymer featuring a higher critical solution temperature, which allows for actuation even in non‐aqueous environments.^[^ ^299^ ^]^

Anisotropic carbon nanotube sheets are another smart material, and can serve as electrothermal actuators (ETAs) to generate large‐strain, multiform movements. Because configurations of carbon nanotubes (CNTs) are conductive and flexible, Li et al.^[^ ^68^ ^]^ proposed polymer ETAs based on heat‐resistant PDMS and CNT paper, which can realize various controllable movements, such as large‐strain bending (>180°) and helical curling (≈630°). They also have a long service life (over 10 000 uses), and their driving voltage is low (20–200 V). Moreover, they are characterized by a faster response speed than many reported ETAs.^[^ ^304^, ^305^ ^]^ As shown in Figure 14C, five fingers based on a CNT‐PDMS double‐layer ETA all bend inward and then unfold.

Programmable stimuli‐responsive actuators are promising for application in CBGs. For instance, Han et al.^[^ ^289^ ^]^ reported a soft gripper based on graphene oxide artificial muscles that consist of an AuNRs@GO/PMMA film with light‐sensitive and wavelength‐selective properties. Via doping with different gold nanorods (AuNRs) with different aspect ratios, three kinds of AuNRs with central absorption wavelengths of 533, 637, and 813 nm were obtained. An artificial finger combined with different sizes of AuNRs at various joints was then fabricated, as shown in Figure 14D, which can implement light‐addressable manipulation.

The precise navigation and cordless delivery of micro‐objects, especially drugs, is another promising application.^[^ ^306^, ^307^ ^]^ One representative example is a flexible micrometer‐scale robot with programmable 3D magnetization. The manufacture of the flexible robot is conducted by the following steps. First, pre‐magnetized hard magnetic particles are mixed with flexible ultraviolet (UV)‐curable materials, and a permanent magnet is then used to generate a magnetic field to precisely reorient all the magnetic particles. After particle reorientation, UV light is applied to selected regions of the substrate to freeze the magnetic particles within those regions. By repeating these steps, a robot with a programmable magnetization can be fabricated. As shown in Figure 14E, a gripper can fold up, roll toward, and pick up the cargo under a specific magnetic field.^[^ ^292^ ^]^ In another study, Li et al.^[^ ^69^ ^]^ designed a pH‐responsive hydrogel‐based soft microrobot, as shown in Figure 14F. The microrobot consists of a hydrogel bilayer structure that includes a 2‐hydroxyethyl methacrylate (PHEMA) layer and a poly (ethylene glycol) acrylate (PEGDA) layer with iron oxide particles (Fe_3_O_4_). The PHEMA layer, as a pH‐responsive gel, is used to hold and release carried drugs in a specific location, and exhibits full trapping motion at about pH 9.58 and unfolding motion at about pH 2.6. Meanwhile, the PEGDA‐with‐Fe_3_O_4_ layer is used to drive the soft microrobot via a magnetic field.

The response to a variety of stimuli is a potential application of soft grippers. Figure 14G presents an example of a smart material responding to an electric field. The tweezer‐shaped gel gripper based on the electrically‐assisted ionoprinting technique can precisely manipulate lightweight objects (0.1–1 g) with a fast response. Via the alternation of positive and negative potentials to the two copper wires, the gel produces an alternating force to close or open the gripper.^[^ ^293^ ^]^

Dong et al.^[^ ^294^ ^]^ reported a small‐scale soft gripper that can respond to humidity. The gripper consists of a graphene oxide/polypyrrole bilayer structure. The graphene oxide layer expands with an increase in humidity due to water absorption, while the polypyrrole layer is almost inert to humidity changes. Consequently, the gripper bends toward its polypyrrole‐layer side. As shown in Figure 14H, the gripper can mimic the claw of a hawk to pick up a cuboid polymer foam (14 × 14 × 7 mm), the weight of which (27.7 mg) is 38 times greater than that of the graphene oxide/polypyrrole gripper (0.73 mg).

Besides, Cheng et al.^[^ ^290^ ^]^ proposed an electrothermal soft gripper based on biomimetic conductive tendrils that are introduced with pre‐strain (400%) during fabrication to form the tendril structure. The helical polyester yarn consists of polyester fibers embedded in conductive tendrils to enhance its reversible contraction actuation ability (with a high spring constant of 2.27 N m^−1^). As illustrated in Figure 14I, the soft gripper is composed of three conductive tendrils and an elastic base. When it is stimulated by Joule heating, the soft gripper can pick up a black polymer foam.

The direct integration of the actuators of creatures with soft grippers is another novel driving mode. Uesugi et al.^[^ ^300^ ^]^ developed an insect muscle‐powered bio‐actuator made from dorsal vessel tissue. The bio‐actuator can contract in response to stimulation, including tensile, thermal, electrical, and chemical stimulation. The maximum contractile force of the bio‐actuator is about 118.1 µN. As shown in Figure 14J, the microgripper is actuated by the contractile force of dorsal vessel tissue.

#### 3.8.2 CTGs Based on Other Smart Materials

Programmable polymers and hydrogel architectures can perform various complex shape deformations, such as self‐twisting caused by inhomogeneous swelling,^[^ ^308^ ^]^ which is induced by water, light, or heating. Many planar‐to‐helical transformations of smart materials have been achieved by learning from and emulating nature.^[^ ^309^, ^310^ ^]^ However, the shape deformation of these smart materials and their predetermined structure represent a one‐to‐one correspondence. In other words, their movement patterns are fixed, and, as a result, their adaptability to objects with different shapes is poor. Additionally, they usually do not have enough loading ability for use in CTGs in daily life.

One example is a soft tendril‐inspired twining‐type gripper, which is composed of programmable polymer‐paper bilayer composites. The polymer‐paper bilayer composite sheet is fabricated with SMP via 3D printing on a rough and porous paper substrate (copy paper with a thickness of 0.1 mm) with different patterns. The filament orientations of the SMP on the paper are the determinant of mechanical performance.^[^ ^77^ ^]^ As shown in Figure 14K, the gripper can produce sequential deformation to grasp the branch like a plant tendril.

## 4 Controlled Stiffness of Soft Grippers

Generally, rigid robotic grippers have rigid limbs and hinge joints, which ensure precise control and structural stability. The existence of the rigid structure allows the manipulator to exert large amounts of force so that heavy objects can be picked up. Soft robotic grippers consist of a continuous or discontinuous soft structure, which ensures both flexibility and compliance. The flexibility and compliance of soft robotic grippers are helpful for improving their grasping efficiency and adaptability to objects with different shapes. Soft robotic grippers also exhibit the advantage of security when interacting with humans. However, compared to rigid robotic grippers, the load capacity of soft robotic grippers is relatively low. Controlling the stiffness of soft grippers is a method that can be implemented to overcome these drawbacks. Soft grippers based on variable stiffness have some of the advantages of both rigid and soft robotic grippers, including flexibility and compliance in the low‐stiffness state, as well as higher structural rigidity and load acceptance in the high‐stiffness state. A stiffness‐controllable structure is usually integrated into grippers as a module. In the low‐stiffness state, adaptive grasping is more comfortable to implement, while in the high‐stiffness state, the gripper can effectively generate sufficient holding force.

The current stiffening strategies include the use of the jamming effect, electrorheology, magnetorheology, low‐melting materials, and SMPs, as shown in **Table** **3**. Electrorheological (ER) and magnetorheological (MR) fluids have a millisecond‐level response time. However, they usually require high currents or high voltages to activate. Low‐melting materials and SMPs have a broad range of modulus variations, and their stiffness can be changed by heating or cooling. The jamming effect is the most common method by which to change stiffness, as the stiffness is controlled by negative pressure, which is safer for operators.

**Table 3:** Stiffness‐controllable strategies and their features

Stiffening strategy	Control methods	Stiffening principle	Response time	Range of modulus variation
Jamming effect	Gas pressure	Friction	0.1–1.1 s^[^ ^311^, ^312^ ^]^	2–100 MPa^[^ ^313^ ^]^
Electrorheological fluids	Electric field	Viscosity change	14 ms^[^ ^314^ ^]^	0.055–0.078 MPa^[^ ^314^ ^]^
Magnetorheological fluids	Magnetic field	Viscosity change	10–170 ms^[^ ^315^, ^316^ ^]^	4.73–62.04 KPa^[^ ^317^ ^]^
Low‐melting materials	Thermal energy	Phase transition	<1 s^[^ ^318^ ^]^	0.0952–860 MPa^[^ ^319^ ^]^
SMPs	Thermal energy	Glass transition	0.5–1 s^[^ ^320^ ^]^	0.01–3 GPa^[^ ^238^ ^]^

### 4.1 Grippers Based on the Jamming Effect

**Figure 15:**
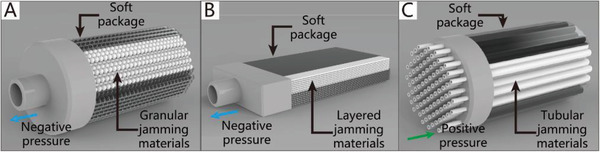
Basic work principles of frequently used jamming objects. A) Granular jamming materials. B) Layered jamming materials. C) Tubular jamming materials.

The jamming effect is the transition of the effector from a liquid‐like to a solid‐like state.^[^ ^321^ ^]^ Generally, soft grippers generate the jamming effect by using the friction of jamming objects in their interior, thereby changing the stiffness. The most frequently used jamming objects include granular materials, layers, and tubular materials, the basic working principles of which are presented in **Figure** **15**. In practical applications, as the pressure in the chamber of the gripper decreases (or increases), the jamming objects squeeze each other to generate considerable friction, thereby improving the structural rigidity. At this point, the entire effector can be regarded as a solid object. After removing the negative pressure, the effector returns to the soft state.

#### 4.1.1 Granular Jamming

**Figure 16:**
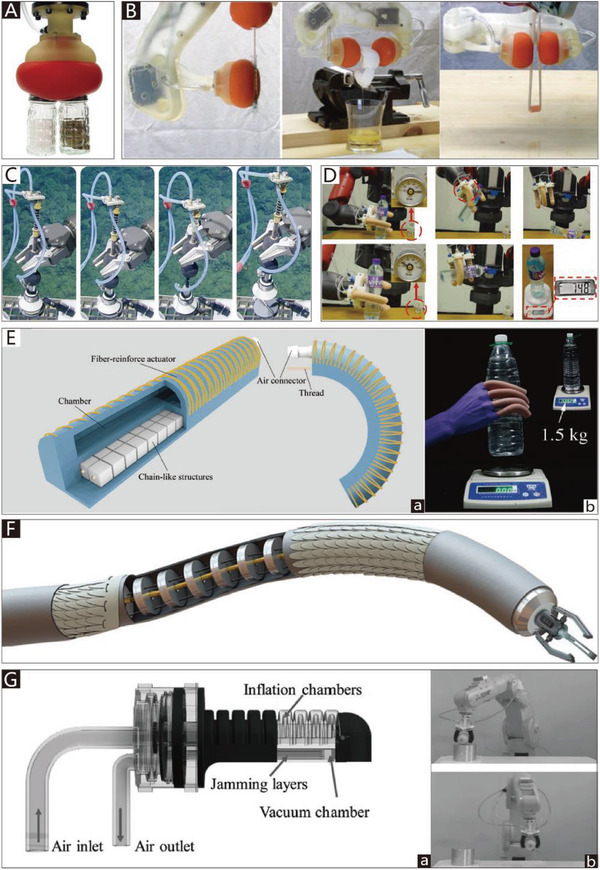
Soft grippers based on jamming effect. A) A universal jamming gripper. Reproduced with permission.^[^ ^311^ ^]^ Copyright 2012, IEEE. B) A two fingers JamHand. Reproduced with permission.^[^ ^324^ ^]^ Copyright 2017, Mary Ann Liebert, Inc. C) A ball‐type jamming gripper as a sea sampling tool. Reproduced with permission.^[^ ^325^ ^]^ Copyright 2017, Mary Ann Liebert, Inc. D) A variable stiffness robotic gripper by granular jamming. Reproduced with permission.^[^ ^326^ ^]^ Copyright 2016, Mary Ann Liebert, Inc. E) A chain‐like granular jamming gripper. Reproduced with permission.^[^ ^327^ ^]^ Copyright 2019, Mary Ann Liebert, Inc. F) A soft arm based on tubular stiffening sheath. Reproduced with permission.^[^ ^328^ ^]^ Copyright 2018, Mary Ann Liebert, Inc. G) A soft gripper integrated layer jamming units. Reproduced with permission.^[^ ^329^ ^]^ Copyright 2019, Mary Ann Liebert, Inc.

In general, a granular jamming structure consists of a soft package and granular materials, which are usually spherical objects such as beans, glass balls, plastic, coffee grounds, and metallic beads.^[^ ^322^ ^]^ With the increase of the vacuum degree, the jamming phenomenon occurs, and the stiffness of the entire structure increases. Granular jamming has been widely applied in the field of soft grippers. For example, Lipson et al.^[^ ^323^ ^]^ described a series of simple passive universal soft grippers based on the jamming of granular materials, which consist of a mass of granular materials encased in an elastic membrane. Compared with traditional multi‐fingered soft hands, which require large numbers of sensors and controllers, the jamming gripper based on a single mass of granular material displays the advantages of highly adaptive ability and straightforward control. Two years later, a new universal soft gripper was designed (**Figure** **16**A). This soft gripper can easily pick up soft objects, flat objects, and other objects with complex geometries, which remains a challenge for universal rigid grippers. Via a combination of positive and negative pressures, the new soft gripper exhibits an increase in reliability of up to 85% and an increase in error tolerance of up to 25%, and its workspace can reach 600 mm.^[^ ^311^ ^]^ In 2017, Lipson et al.^[^ ^324^ ^]^ reported the JamHand, which can achieve fundamental dexterous manipulations and precision grasps. The JamHand has two digits, including a “thumb” and a “finger.” The thumb can be rotated around its base, and the finger can be moved by a four‐bar linkage. The fingertips of the thumb and finger contain a large amount of granular material that can be stiffened or softened by controlling the internal air pressure. The JamHand can achieve multiple precise motions, such as operating a syringe, cracking an egg, and using chopsticks, as shown in Figure 16B.

Another application of the ball‐type jamming gripper is as a sea‐sampling tool, which can be used in the deep sea wherein the ambient pressure exceeds 100 atmospheres. Different from the traditional granular jamming gripper, the jamming gripper was developed by Licht et al.^[^ ^325^ ^]^ is composed of a latex balloon filled with a mixture of freshwater and glass beads with a diameter of about 200 µm. Using freshwater, rather than gas, allows for the operation of the soft gripper with a closed‐loop fluid system. The grasping process is presented in Figure 16C; the latex balloon gripper moves downward under the weight of the apparatus and lead weight, and then conforms to the object. Meanwhile, the water in the latex balloon is removed to reach the desired jamming pressure. Finally, the object is picked up with the movement of the gripper apparatus.

While spherical jamming grippers can grasp objects with complex geometries, their spherical shape makes it difficult to pick up objects without noticeable sharp edges. To solve this problem, Wei et al.^[^ ^326^ ^]^ proposed a soft variable stiffness anthropomorphic gripper that has the advantages of both a granular jamming gripper (firm holding) and soft gripper (dexterous grasping). Before grasping an object, the robotic gripper is highly compliant so that it can adapt to the shape of the target object, and its grasping reliability is therefore enhanced. After grasping an object, the gripper is stiffened by granular jamming to freeze its current position, which enhances the load capacity of the gripper. As shown in Figure 16D, the increased stiffness can allow the gripper to grasp a bottle of water weighing 750 g, which is a heavy load for soft grippers.

Soft and stiffness‐controllable instruments have also been used in the medical field. Cianchetti et al.^[^ ^330^ ^]^ reported the STIFF‐FLOP arm for minimally invasive surgery, the three fluidic chambers of which are equally spaced in a radial arrangement. When the atmospheric pressure of the three channels is 0.65 bar, the force of the arm can reach 47.1 N. By changing the pressure in the three fluidic chambers, the STIFF‐FLOP arm can perform different motions. The arm can also readjust its stiffness according to the surgical environment and organs to accomplish specific surgical tasks. The stiffness of the arm is controlled by a stiffening chamber, which is composed of a latex membrane filled with coffee powder and mounted in the center of the arm.

In addition to the traditional vacuum‐based granular jamming grippers, Jiang et al.^[^ ^327^ ^]^ reported a chain‐like granular jamming gripper that can instantly achieve a broad range of stiffness variation. Exploiting the use of threads, hexahedral granules are combined to form a chain‐like jamming structure, as shown in Figure 16E(a), which can achieve a stiffness variation range of as much as 50.7‐fold. Subsequently, an anthropomorphic hand based on the chain‐like jamming structure was developed, which is not only able to pick up a heavy load in the rigid state (3.52 kg with a hook gesture), but is also versatile enough to accomplish complex grasping tasks in the compliant state. Furthermore, the anthropomorphic hand is inexpensive and allows for a fast response. The gripper can hold up a 1.5 kg bottle of water, as shown in Figure 16E(b). In addition, the significance of membranes for the granular jamming system was researched by Jiang et al.,^[^ ^331^ ^]^ who found latex and nitrile to be versatile materials. Vinyl and vitrile were found to be quite varied between the load scenarios, and performed better than latex in some scenarios, while worse in others. Polythene was found to consistently achieve the highest Young's modulus values.

#### 4.1.2 Layer and Tubular Jamming

Layer jamming is another approach to achieve the jamming effect. Compared with granular jamming, it has advantages in terms of cubage and weight. One representative example is a new variable compliance structure based on mechanical layer jamming. This new approach does not require fluidic actuation like granular jamming; instead, layers of jamming structures, which consist of scale layers with protruding flaps, are brought together and loosened by the direct mechanical actuation of a braided mesh. The compact and light structure provides a more effective mechanical approach to layer jamming, which is based on controlling the friction between special structures of compressed layers. Consequently, the stiffness of the gripper is directly proportional to the amount of friction between the compressed layers. In its loosened state, the movements of bending, contraction, and extension are achievable.^[^ ^332^ ^]^

Exploiting the layer jamming approach in the same way, Langer et al.^[^ ^328^ ^]^ developed a tubular stiffening sheath for continuum soft arms to change the stiffness in a certain configuration. By continuously weaving the wire through a lower row of holes in a double‐sided flap pattern, a reversible stiffening sheath with layer jamming capability can be fabricated. Then, the sheath is mounted between the inner and outer membranes, as shown in Figure 16F. Depending on the requirements of the task, the soft arms can change between the compliance and stiffness states by adjusting the vacuum condition of the arm. In an array of experimental evaluations, it was ultimately proven that the performance of the layer jamming structure based on a reversible stiffening sheath is superior to the performance of the traditional granular jamming structure.

Different from the jamming structures with scale or sheath layers mentioned previously, Zhu et al.^[^ ^329^ ^]^ proposed a soft gripper that contains two identical fingers, each of which contains a parallel integrated‐layer jamming unit composed of a laminated structure and a vacuum chamber, as depicted in Figure 16G(a). The soft finger implements bending movement by inflating the soft body through the air inlet, and the jamming unit increases the stiffness by vacuuming the chamber through the air outlet, which can guarantee grasping robustness when the soft gripper moves at a maximum speed of 1.5 m s^−1^ and with an acceleration of up to 8 m s^−2^, as shown in Figure 16G(b).

Besides, Miller‐Jackson et al.^[^ ^333^ ^]^ proposed a new and practical tubular jamming approach with a stiffening effect, which is adaptable to the motion of actuators and lightweight, and can be implemented with minimal equipment. The fabrication process of the tubular jammed beam is simple; only the tubules need to be packed into the soft sleeve to form a tubular jammed beam. The tubules are pressurized so that they inflate to fill the containing structure, resulting in the production of the jamming effect between tubules (the contact of the tubules with each other produces large friction forces to inhibit their movement). Compared with granular jamming and layer jamming, tubular jamming does not require an additional vacuum pump; the jamming effect is produced by positive pressure, which is also used by soft grippers. As a typical jamming structure, the tubular jammed beam has an advantage in terms of its bending stiffness; under the maximum pressure, the maximum bending stiffness of the tubular jammed beam is nearly three times that of traditional SPA beams. Therefore, the tubular jammed beam requires lower supply pressure to achieve the same performance as its traditional counterpart.

The reachable stiffness of soft grippers is dependent on the overall volume used for jamming, the applied pressure, the membrane, and the jamming materials.^[^ ^328^ ^]^ Future research regarding the jamming effect could focus on increasing the volume used for jamming under the prerequisite of retaining the volume of grippers, as well as finding a membrane with better mechanical performance to meet a higher negative pressure. Moreover, the jamming material may be a new break‐through point. Small‐volume, lightweight jamming structures are expected to replace the existing granular jamming, layer jamming, and tubular jamming structures in future applications.

### 4.2 Electrorheological and Magnetorheological Fluids

**Figure 17:**
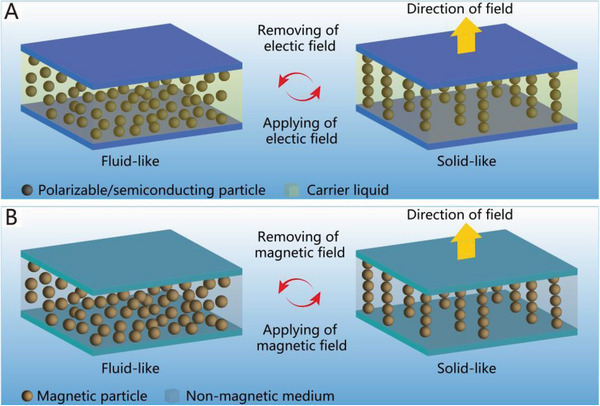
Schematic diagrams of ER fluid and MR fluid. A) Basic work principles of ER fluid. (Modified and redrawn.) Reproduced with permission.^[^ ^337^ ^]^ Copyright 2019, Royal Society of Chemistry. B) Basic work principles of MR fluid. (Modified and redrawn.) Reproduced with permission.^[^ ^338^ ^]^ Copyright 2010, Royal Society of Chemistry.

ER fluids are colloidal suspensions (including polarizable particles with sizes of 0.1–100 µm), the viscosity and yield stress of which are strongly affected by the electric field intensity.^[^ ^334^ ^]^ As shown in **Figure** **17**A, when an electric field is applied (0.5–3 kV mm^−1^), ER fluids can transform from a fluid‐like to a solid‐like state within a millisecond.^[^ ^335^ ^]^ However, it is worth noting that ER fluids usually work at a relatively high voltage of 1–5 kV, which is a risk factor for service robots that require safety.^[^ ^336^ ^]^ Therefore, ER fluids are rarely used for soft grippers.

An exception was proposed by Tonazzini et al.,^[^ ^314^ ^]^ who designed a flexible fluidic system based on ER fluids that can act as an arm module in a soft gripper. The flexible fluidic system consists of four flexible fluidic actuators, each of which includes an inlet valve and discharge valve, and an electric field is applied to change the viscosity of the fluid so that the pressure inside the actuators is different. The elongation of the four flexible fluidic actuators causes the bending or elongation of the system.

MR fluids are a type of smart fluid, the rheological behavior of which can be changed under the action of a magnetic field.^[^ ^339^ ^]^ The basic working principle of MR fluid is shown in Figure 17B. MR fluids usually consist of oil and micrometer‐ or nanometer‐scale ferromagnetic particles.^[^ ^315^ ^]^ They also contain a surfactant to prevent ferromagnetic particles from forming sediment in the oil. The viscosity of the MR fluid can increase by 10^5^–10^6^ times via the application of a magnetic field, and the response time of the MR fluids is swift, on the order of milliseconds.^[^ ^340^ ^]^ Typically, MR fluids can produce more considerable shear stress than dispersed ER fluids under the same electric field. Therefore, MR fluids are more popular than ER fluids in the field of soft grippers.

**Figure 18:**
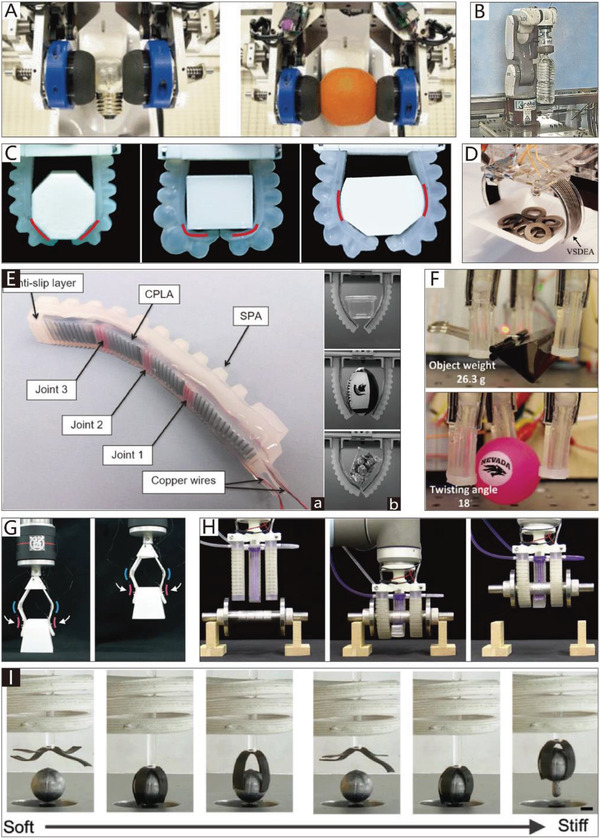
Stiffness‐controllable soft grippers based on magnetorheological fluids, low‐melting materials, and shape memory materials. A) A universal parallel gripper using MR fluid. Reproduced with permission.^[^ ^342^ ^]^ Copyright 2017, IEEE. B) A universal ball‐type robot gripper using MR fluid. Reproduced with permission.^[^ ^343^ ^]^ Copyright 2016, World Scientific. C) A pneumatic soft gripper with LMPA stiffness‐controllable structure. Reproduced with permission.^[^ ^344^ ^]^ Copyright 2018, IOP Publishing, Ltd. D) A variable stiffness gripper consisted of DEA and LMPA. Reproduced with permission.^[^ ^345^ ^]^ Copyright 2015, IEEE. E) A soft gripper with stiffness and shape modulation. Reproduced with permission.^[^ ^346^ ^]^ Copyright 2019, Mary Ann Liebert, Inc. F) A three‐fingered pneumatic soft gripper contained ‘‘programmable’’ ligaments. Reproduced with permission.^[^ ^347^ ^]^ Copyright 2017, Mary Ann Liebert, Inc. G) An SMA‐based soft gripper with SMP joints. Reproduced with permission.^[^ ^348^ ^]^ Copyright 2017, Mary Ann Liebert, Inc. H) A three‐fingered gripper based on SMP variable stiffness module. Reproduced with permission.^[^ ^349^ ^]^ Copyright 2019, John Wiley and Sons. I) A soft gripper based on magnetic SMP. Reproduced with permission.^[^ ^350^ ^]^ Copyright 2019, John Wiley and Sons.

To avoid hard contact with traditional rigid grippers, which usually results in damage to delicate natural food products like tomatoes, strawberries, and grapes, Pettersson et al.^[^ ^341^ ^]^ developed an MR robotic gripper with the features of compliance and variable stiffness. The gripper consists of two fingers, one of which is fixed, and the other of which is driven by a belt‐driven transmission system. The fingers are mounted with electromagnets that are powered with a 24 V DC voltage. MR fluid‐filled pouches are fixed on the two fingertips to change the stiffness of the gripper. In the gripping process, the MR fluid‐filled pouches are compliant to adapt to the target objects. In the transporting process, the pouches are stiffened by a magnetic field to freeze their current position. Using a similar principle, Tsugami et al.^[^ ^342^ ^]^ developed a universal parallel gripper that uses reformed MR fluid, which can pick up a light bulb and an orange, as shown in **Figure** **18**A. The gripper also has two fingertips constructed with an elastic membrane enclosing the MR fluid. The difference of this gripper lies in the method used for the control of the viscosity of the reformed MR fluid; the existing method of changing the magnetic field intensity is replaced by moving a permanent magnet.

The MR fluid has many advantages, but its low hardness upon solidification has been the primary reason for its limited range of applications. To overcome this deficiency, Nishida et al.^[^ ^343^ ^]^ proposed a universal ball‐type robotic gripper that uses an electromagnet and a reforming MR fluid, which is made by adding nonmagnetic particles to normal MR fluid (Figure 18B). Along the lines of magnetic flux, the ferromagnetic particles form many columnar structures when a magnetic field is applied, and the nonmagnetic particles simultaneously squeeze between these structures to transform the MR fluid into a solid. This reforming MR fluid resolves several issues faced by normal MR fluid, such as its low hardness upon solidification and comparatively large specific gravity. Under the same magnetic flux density, the solidification force of the reforming MR fluid is more than twice that of normal MR fluid. Moreover, its specific gravity is only about half as large as that of normal MR fluid. Due to the increase of hardness upon solidification, the maximum gripping force of the ball‐type soft gripper can reach 52.84 N.

Both ER and MR fluids have a millisecond‐level response time, and are therefore faster than their competitors. However, high voltages are required to activate ER fluids. Future work for the use of ER fluids in soft grasping systems will include the reduction of the activating voltage and the discovery of suitable protective measures. Regarding MR fluids, their low hardness upon solidification remains an essential factor that hampers their implementation. Although stronger magnetic fields could be implemented by increasing the electric current, the heat generation of the system will increase sharply as a result. Improving the energy conversion efficiency of the electromagnetic system and the absolute stiffness of MR fluid are future development directions.

### 4.3 Low‐Melting Materials

Low‐melting materials include low‐melting‐point alloys (LMPAs) and other materials that can undergo phase changes from a solid to liquid by heating. Conversely, they can revert to the solid‐state by cooling. The phase change of low‐melting materials can be exploited to obtain variable stiffness structures. They are usually embedded in soft shells to avoid being drained away in the liquid state.

In general, the melting point of LMPAs can be changed by integrating different metallic materials to meet the various needs of stiffness‐controllable structures. For example, particles of Field's metal, which is a eutectic, fusible alloy of bismuth, indium, and tin with a melting point of ≈62.5 °C, can be quickly melted in hot water. Based on Field's metal particles, Buckner et al.^[^ ^351^ ^]^ reported a soft actuator with the advantages of variable stiffness and variable stretchability. The actuator consists of two layers, including a pneumatically actuated layer and an FMEpoxy material layer, which is fabricated by mixing Field's metal particles into an epoxy matrix. When the FMEpoxy material layer is heated, the soft actuator can be inflated and curled up under the drive of the pneumatically actuated layer. After the FMEpoxy is cooled in the bending state, the soft actuator has a bending configuration in that the FMEpoxy material layer is in a state of high stiffness. Using a similar design principle, Yoshida et al.^[^ ^352^ ^]^ reported a pneumatic bending finger integrated with an LMPA‐based variable stiffness skeleton (Sn 8.3%, Bi 44.7%, Pb 22.6%, Cd 5.3%, and In 19.1%), which can bend at specific points (by selecting different points of the LMPA to melt) and maintain its bent shape without a pressure supply. The local stiffness of the finger can be altered by melting or hardening the LMPA with Joule heating applied via integrated Ni–Cr wires.

The cooling of LMPAs at room temperature requires a significant amount of time. To improve the working efficiency of actuators, Hao et al.^[^ ^344^ ^]^ developed a eutectic alloy‐infused soft actuator. Different from the soft actuator previously described, in addition to the use of an LMPA (32.5% Bi, 51% In, and 16.5% Sn by weight) to change the stiffness and EGaIn (75.5% Ga and 24.5% In by weight) to sense the curvature information of the actuator, a water‐cooling system is mounted in the actuator to quicken the dynamic response speed of the actuator. After heating the LMPA with a Ni–Cr wire, water is pushed into the actuator to increase the cooling rate. By switching its motion patterns between “rigid” and “compliant,” the soft gripper can pick up objects with various shapes, as shown in Figure 18C. The maximum load of the gripper is 280 g, which is 20 times the weight of a single actuator.

In addition to the combination of SPAs and LMPAs discussed previously, the DEA is an alternative actuator. Shintake et al.^[^ ^345^ ^]^ developed a variable stiffness gripper that consists of a DEA and an LMPA embedded in a silicon substrate. The DEA is used to generate a bending actuation, while the LMPA is used to provide controllable stiffness between the compliant and rigid states via Joule heating. In the rigid state, a fixed shape can be maintained without the help of the DEA. Moreover, its spring constant is 90 times that of the LMPA‐free actuator. As shown in Figure 18D, the gripper with an active component weight of ≈2 g can hold an object with a mass of 11 g in the rigid state.

Compared with LMPAs, low‐melting nonmetallic materials are usually lightweight, which makes them more suitable for the miniaturization of the entire system. An example is a soft finger with a variable stiffness skeleton fabricated by a 3D‐printed conductive polylactic acid (CPLA) material. The stiffness of this material can be conveniently controlled via Joule heating, and its glass transition temperature is 55 °C, at which its Young's modulus is 60% at room temperature.^[^ ^346^ ^]^ A fabricated soft finger prototype with an embedded CPLA layer and an anti‐slip layer is shown in Figure 18E(a). The gripper based on the finger prototypes can pick up multiple objects, as shown in Figure 18E(b), and its maximum load is 800 g.

As a low‐melting nonmetallic material, a conductive propylene‐based elastomer‐PDMS composite has also been applied in a three‐fingered pneumatic soft gripper as ‘‘programmable’’ bionic ligaments. Each finger has three bionic ligaments that undergo a stiffness change when Joule heating is applied. Depending on which ligaments are heated, the fingers of the gripper will bend inward to pick up an object, bend laterally to twist it, or bend outward to release it. All the gripper motions are generated with a single pneumatic pressure source. An activation–deactivation cycle of the gripper can be completed within 15 s.^[^ ^347^ ^]^ The gripper can pick up a 26.3 g paper clip and twist a 2.1 g ping‐pong ball, as shown in Figure 13F.

Future studies of low‐melting materials should explore methods by which to minimize the cooling time. Natural cooling requires a significant amount of time, and some soft prototypes take more than 60 s to cool to room temperature.^[^ ^318^ ^]^ The integration of a water‐ or air‐cooling system in the gripper could be a way to speed up cooling. Furthermore, the existing low‐melting materials are mostly subjected to Joule heating, which requires wires to transmit power. The application of vortex heating or other non‐contact heating methods can remove the limitations imposed by wires, which are unfavorable to miniaturization and usually introduce inconvenience.

### 4.4 Shape Memory Materials

Shape memory materials include SMPs and SMAs, which exhibit stiffness variation when the applied thermal energy changes. However, the stiffness change of SMAs is relatively small, and the Young's modulus remains high (21‐41 GPa for martensite and 30–83 GPa for austenite),^[^ ^353^ ^]^ which limits the stiffness adjustment module of SMAs in a soft gripper.^[^ ^225^ ^]^ In fact, SMAs are a better choice as an actuator. Conversely, thermally activated SMPs are suitable as a stiffness‐tunable material. Their working principle is shown in Figure 10A; after heating, SMPs soften when the temperature increases past a certain transition temperature. After cooling, the SMPs can hold a temporary shape with a higher stiffness. Their stiffness can reversibly change from a few MPa (a soft rubbery state) to a few GPa (a stiff glassy state).^[^ ^233^ ^]^

Figure 18G presents an example of an SMA‐based soft gripper with SMP joints, which can independently achieve an approximately 55‐fold changeable stiffness by Joule heating. By integrating a changeable stiffness module (SMP joints), the three‐fingered gripper is applicable to objects with a broad range of weights. Moreover, its maximum grasping force is increased by ≈10 times. The time required for the SMP joints to change from the high‐stiffness state to the low‐stiffness state has been determined as 40 s. As there is no cooling system, the inverse process takes a longer amount of time, roughly 240 s.^[^ ^348^ ^]^

The heat dissipation of SMPs usually takes a great deal of time. To overcome this, Zhang et al.^[^ ^349^ ^]^ proposed a hybrid multi‐material 3D‐printed soft gripper integrated with a Joule‐heating circuit and a fluidic‐cooling microchannel. The rapid and effective heating and cooling allow the stiffness‐tunable actuator to complete a softening‐stiffening cycle within 32 s. By integrating an SMP layer into the actuator, its stiffness is enhanced by up to 120 times without sacrificing its compliance and flexibility, which allows the actuator to grasp and lift objects with arbitrary shapes. The object weight span of the soft actuator ranges from less than 10 g to up to 1.5 kg. As shown in Figure 18H, the three‐fingered gripper based on soft actuators can grasp and lift a dumbbell weighing 1.5 kg. In addition to its stiffness adjustment, the shape memory effect of the SMP can simultaneously be applied in a soft gripper. One representative example is a robotic finger developed by Yang et al.^[^ ^354^ ^]^ The finger is composed of a substrate and a SPA, which are made of 3D‐printed multi‐materials. Inspired by the physiological structure of human fingers, the substrate has three SMP joints and four polylactic acid connecting pieces. When the SMP joints are heated above the transition temperature, they exhibit very little stiffness, allowing the soft actuator to drive the finger to bend easily around the joints. By exploiting the shape recovery stress of the SMP, the finger can quickly be restored to its initial state without the external force of the soft actuator. Because the motion of each joint can be individually controlled, the mobility of the finger is very flexible.

As a stiffness‐controllable material, the method of Joule heating is mainly adopted for thermally activated SMPs, which requires wires to transmit energy. Different from the existing methods of stiffness adjustment, Ze et al.^[^ ^350^ ^]^ developed a soft gripper based on a magnetic SMP that is heated by an untethered method to change its stiffness, which is called the magnetothermal effect. The magnetic SMP is integrated with NdFeB and Fe_3_O_4_ particles; the NdFeB particles are used as actuation particles driven by the actuation magnetic field, and the Fe_3_O_4_ particles are used to heat the magnetic SMP by applying a high‐frequency AC magnetic field. When the temperature of the magnetic SMP is higher than its glass transition temperature, its modulus decreases significantly. Then, a small actuation magnetic field can easily bend the magnetic SMP. As shown in Figure 18I, via the actuation magnetic field and the heating magnetic field, the magnetic SMP gripper can grasp a lead ball (49 times heavier than the soft gripper) in the low‐stiffness state, and can lift it in the high‐stiffness state.

For further developments, a challenge will be to decrease the time of the cooling phase, like that for low‐melting materials. Researchers have begun to integrate fluidic cooling microchannels into actuators to enhance the response speed.^[^ ^349^ ^]^ Furthermore, the untethered heating method is a promising technology for shape memory materials in stiffness‐controllable applications. The shape memory effect and the mechanical properties of shape memory materials should be paid more attention, especially for soft grippers with shape memory materials that can be restored to their initial state by the shape memory effect.

## 5 Conclusions and Perspectives

Grippers and manipulators play crucial roles in the interaction process between creatures and soft robots and their environments. Rigid gripper technologies applied in the industrial and anthropomorphic robot fields have become mature. In recent years, due to the requirements of healthcare, cooperative human assistance, fragile product processing, agricultural products, and food processing, the next generation of grippers with the advantages of friendly operation, high compliance, and compatibility has been developed. As a new bridge between robots and operated objects, soft grippers can complete grasping and operation tasks via adhesion or friction. In this review, an overview of soft grippers was provided.

Since the first appearance of soft grippers, researchers have never ceased to learn from and emulate their counterparts in nature. Thus far, creatures with the ability to grasp and manipulate objects have paved the way in terms of their flexibility and response speed. Inspired by the grasping and operation modes of creature grippers, a novel classification method of soft grippers was proposed based on their movement modes, and includes NBGs, CBGs, and CTGs. Their respective characteristic features were described in Section 2 of this article. In addition, soft actuators are crucial to soft grippers, and are usually integrated into the body of grippers. While providing motive power, they must be compliant enough to adapt to the deformation of grippers. In Section 3, soft grippers, classified according to the type of soft actuator they employ, were discussed. Finally, focus was placed on stiffness control strategies that can overcome some shortcomings of soft grippers, such as their inferior load capacity. Their advantages and limits were also analyzed.

Traditional soft actuators, such as SPAs and cable‐driven actuators, are becoming popular and mature. Moreover, in the field of soft grippers, some emerging soft actuators, including SMPs, SMAs, DEs, LCEs, IPMCs, and other smart materials, have been widely applied. However, the intrinsic bending characteristics of these actuators make them more prone to application in CBGs. Regarding the other soft grippers, especially CTGs, some emerging soft actuators face many challenges in the implementation of complex and flexible motions. Furthermore, a limited gripping force is another defect of some emerging soft actuators, especially for stimuli‐responsive smart materials. A future development trend of soft grippers will be to fabricate advanced soft actuators with a sufficient gripping force, response speed, control precision, and adaptability.

Another significant trend is the combination of grippers and adhesives. In nature, a typical example is the octopus, whose tentacles are covered with suction cups that can help it tightly grasp objects in seawater. Inspired by the octopus, suction cups can be integrated into grippers to improve their ability to pick up objects with smooth surfaces. For instance, a soft gripper combined with tendon‐driven actuators and suction cups could achieve better grasping performance.^[^ ^50^ ^]^ In addition, vacuum adhesion, electroadhesion, gecko adhesion, and capillary adhesion could be combined with grippers to produce an ideal grasping effect.

Many soft grippers are currently used outside laboratory conditions; they serve in the fruit and vegetable industry, flexible exoskeletons, and health services, and help researchers collect aquatic mollusk samples. However, most of the existing soft grippers are limited by their soft materials and soft actuators for application in high‐temperature, high‐pressure, cold, and corrosive environments to meet the demands of research in extreme environments, such as the exploration of space, the deep ocean, and polar regions. Soft grippers usually age rapidly and fail when they work in an extreme environment without any protective measures. Thus, learning from creatures who live in extreme environments may be one way to overcome these problems. An example is a soft gripper based on an anti‐freezing polymeric organic hydrogel actuator (a hydrogel with a glycerol‐water binary solvent). Its anti‐freezing mechanism, in which glycerol is applied to prevent the body fluid from freezing at subzero temperatures, was inspired by invertebrates.^[^ ^355^ ^]^

After years of exploration and development, the performance of soft grippers has been considerably improved. However, compared with their counterparts in nature, there is a significant margin for further improvement. In addition to the limitations of soft actuators mentioned previously, the insufficient capacity to perceive the surrounding environment may be another obstacle. Limited by flexible sensor technology, most previous studies have focused on the perception of pressure and deformation,^[^ ^356^, ^357^, ^358^ ^]^ whereas natural components, such as human skin, can perceive the temperature distribution, the moisture content, and even the roughness of objects. Furthermore, the perception of environmental changes can arouse a series of physiological reactions, such as perspiration. The time of the peak power output can be prolonged by a thermoregulation behavior, which can be used in hydrogel bio‐inspired grippers.^[^ ^359^ ^]^ If researchers can learn from the ability of creatures to obtain information about their environments and react to the perceptions of moisture content, surface texture, and temperature distribution, the application future of soft robotic grippers will be capacious.

## Conflict of Interest

The authors declare no conflict of interest.
